# Genome-Wide Quantitative Analysis of Histone H3 Lysine 4 Trimethylation in Wild House Mouse Liver: Environmental Change Causes Epigenetic Plasticity

**DOI:** 10.1371/journal.pone.0097568

**Published:** 2014-05-21

**Authors:** Angelika G. Börsch-Haubold, Inka Montero, Kathryn Konrad, Bernhard Haubold

**Affiliations:** 1 Department of Evolutionary Genetics, Max Planck Institute for Evolutionary Biology, Plön, Germany; 2 Institute for Evolution and Ecology, University of Tübingen, Tübingen, Germany; 3 Cologne Center for Genomics, University of Cologne, Köln, Germany; Ludwig-Maximilians-Universität München, Germany

## Abstract

In mammals, exposure to toxic or disease-causing environments can change epigenetic marks that are inherited independently of the intrauterine environment. Such inheritance of molecular phenotypes may be adaptive. However, studies demonstrating molecular evidence for epigenetic inheritance have so far relied on extreme treatments, and are confined to inbred animals. We therefore investigated whether epigenomic changes could be detected after a non-drastic change in the environment of an outbred organism. We kept two populations of wild-caught house mice (*Mus musculus domesticus*) for several generations in semi-natural enclosures on either standard diet and light cycle, or on an energy-enriched diet with longer daylight to simulate summer. As epigenetic marker for active chromatin we quantified genome-wide histone-3 lysine-4 trimethylation (H3K4me3) from liver samples by chromatin immunoprecipitation and high-throughput sequencing as well as by quantitative polymerase chain reaction. The treatment caused a significant increase of H3K4me3 at metabolic genes such as lipid and cholesterol regulators, monooxygenases, and a bile acid transporter. In addition, genes involved in immune processes, cell cycle, and transcription and translation processes were also differently marked. When we transferred young mice of both populations to cages and bred them under standard conditions, most of the H3K4me3 differences were lost. The few loci with stable H3K4me3 changes did not cluster in metabolic functional categories. This is, to our knowledge, the first quantitative study of an epigenetic marker in an outbred mammalian organism. We demonstrate genome-wide epigenetic plasticity in response to a realistic environmental stimulus. In contrast to disease models, the bulk of the epigenomic changes we observed were not heritable.

## Introduction

Adaptation to environmental change requires metabolic responses that maintain homeostasis. Such responses are generated by fast regulation of gene expression through transcription factor binding, and by slow modulation of chromatin structure through epigenetic marks. Studies on cell lines or inbred model organisms have uncovered how various epigenetic settings contribute to cellular activity, and an epigenetic code that determines chromatin accessibility has been proposed [1], [2]. This code comprises DNA methylation states, histone modifications, and non-coding RNA molecules, which together subdivide the genome into active, poised, and silent regions [3]–[5].

Adaptive epigenetic responses can be induced by nutrition, temperature, population density, and stress [6]. These factors have a cumulative effect throughout the life-time of an organism. For example, 50-year-old monozygotic twins differ substantially in epigenetic marks, whereas 3-year-old twins do not [7]. Further examples of induced epigenetic changes in outbred mammals come from humans exposed to hunger, toxins, or psychological stress [8], [9]. Important in an evolutionary context, however, are observations of non-Mendelian inheritance of acquired traits in various organisms, including mammals [10]–[13]. For example, historical starvation periods in humans have lead to paternally inherited greater cardiovascular mortality and diabetes risk through epigenetic changes at the imprinted locus INS-IGF2-H19 [14]–[16].

Experiments with manipulated feeds are perhaps the most widely used approach to unraveling the molecular mechanisms of adaptive epigenetic changes in mammals (for recent reviews, see [17] and [18]). For example, feeding low protein diets to pregnant rats leads to fetal growth retardation and an increased risk of obesity in the adult offspring. This is due to changes in DNA methylation at the promoters of a few regulatory and metabolic genes [19]–[22]. Moreover, the intrauterine effects of protein-restricted diets on the hepatic promoters of the glucocorticoid receptor *Nr3c1* and of the peroxisome proliferator-activated receptor alpha (*Ppara*) persist into the F2 generation [23]. Changes of phosphoenolpyruvate carboxykinase promoter methylation were even found in F3 [22]. Potentially even more significant is that male rats on a high-fat diet have diabetic female offspring characterized by specific cytosine hypomethylation [24]. Similarly, metabolic changes in the liver of mouse offspring correlate with the hypermethylation of several CpG islands in a putative enhancer region upstream of *Ppara* [25]. Other examples of experimentally induced transgenerational epigenetic inheritance in mammals include responses to toxins [26]–[29], and to early-life stress [30], [31].

It has long been speculated that epigenetic inheritance would contribute to an organism’s evolvability because epigenetic change can be much faster than changes in allele frequencies [32]–[36]. Unfortunately, molecular studies of epigenetic inheritance are limited to inbred animals, because their lack of genetic variation helps in the interpretation of inherited effects. This means, however, that we do not know how wild-type epigenomes respond to environmental change. Moreover, the environmental change imposed on laboratory animals is typically either toxic or disease-causing. To put epigenetic adaptation in an ecological context therefore requires experimental work where outcrossing organisms are exposed to realistic habitats [37]–[39].

**Figure 1:**
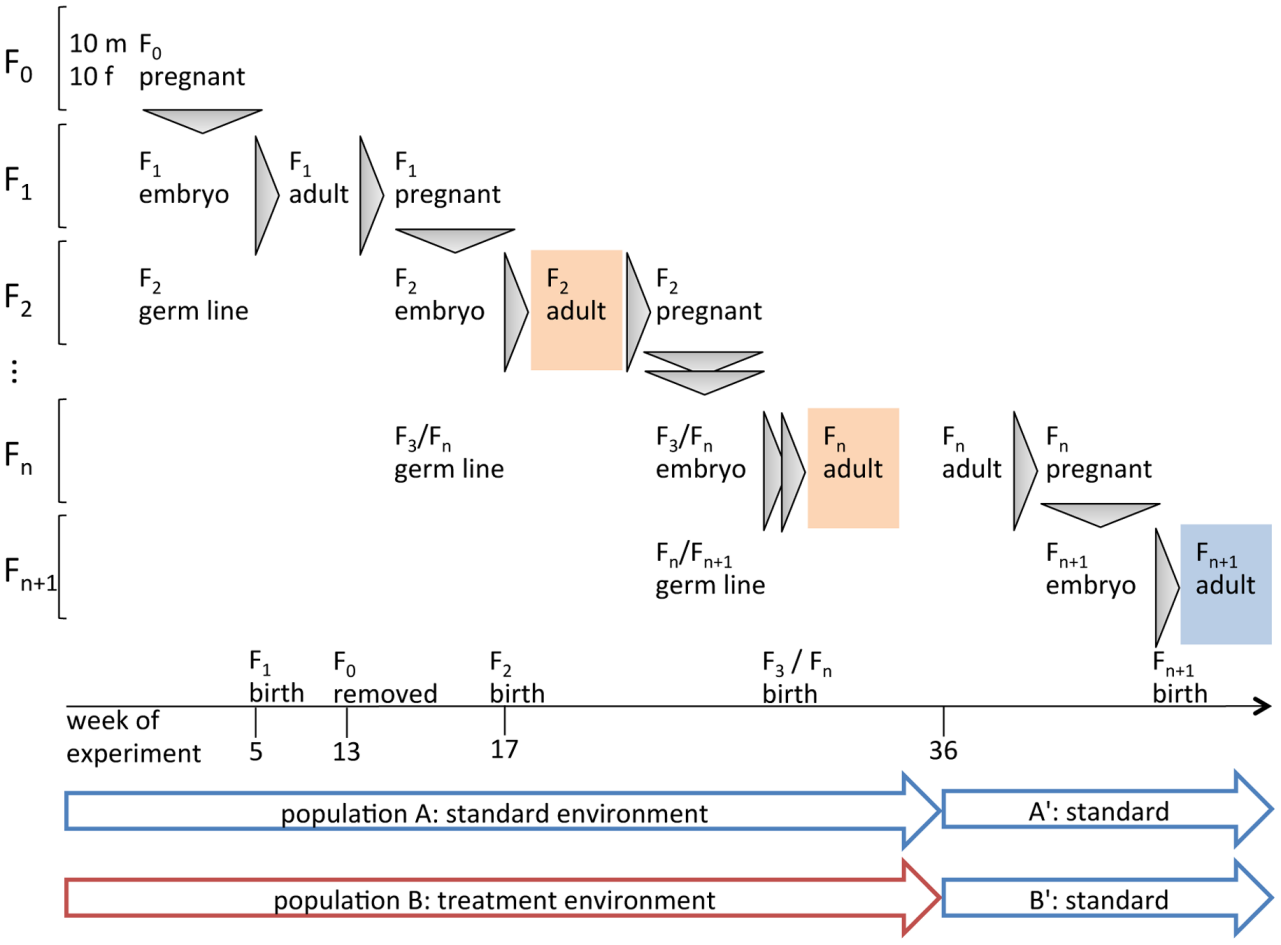
Experimental setup. Mouse populations A and B were started with 10 males (m) and 10 females (f) each (F_0_). Siblings from out-bred wild house mice were evenly distributed to the two enclosures to ensure similar genetic diversity. Population A is the control (standard breeding conditions), population B the treatment group (high-energy diet, prolonged light period and, from week 17 onwards, higher mouse density). After week 13, founders (F_0_) were removed from the experiment so that only animals that were born in the enclosures produced further offspring. After 36 weeks, eight young males of generations F_2_ and higher were chosen for ChIP-Seq (orange shades). At the same time, young males and females (body weight 14–15 g) were transferred to cages and bred under standard conditions. Their offspring (F_n+1_) are populations A′ and B′ of which eight males were selected for ChIP-Seq (blue shade).

To better understand the role of the epigenome in adaptation to natural habitats, we quantified epigenetic responses in wild-caught house mice (*Mus musculus domesticus*) exposed to a mild environmental change. Mice were left in two semi-natural enclosures similar to those used by Crowcroft [40], [41] to produce several generations of offspring. Population A was kept under the standard conditions that we use in our breeding facility. To simulate summer with better food and more light, population B was given a high-energy diet and was kept at longer day-times. After eight months, young animals represented generations F_2_ and higher (Figure 1). This means that the male and female gametes from which they grew had developed in the environment affected by the experiment. At this time point, we captured eight healthy young males for epigenetic analysis. In addition, four- to five-week old animals (F_n_ in Figure 1) were brought into cages and kept under standard conditions. Their offspring, populations A′ and B′ (generation F_n+1_), were used to assess the stability of epigenetic changes in the absence of the inducing environment.

We chose liver for epigenetic analysis, because metabolism converges there, as well as several pathways of nutrient transport, uptake, and storage. In addition, the liver is the target for many steroid and peptide hormones released from the hypothalamus. Environmental or social factors that initially affect the brain could also signal to liver. To scan modifications across the genome, we used the technique of selective chromatin-immunoprecipitation followed by high-throughput sequencing (ChIP-Seq; [42]–[44]). We quantified histone 3 trimethylation at lysine 4 (H3K4me3), because we expected that a strong activating mark [45]–[47] would be most likely to respond to an increase in energy and light. In fact, we found that H3K4me3 marks changed at multiple loci and demonstrate that the epigenome reacts in a directed way to even mild environmental changes. In contrast to transgenerational inheritance after drastic treatment, few if any of these changes persisted after the environmental signal was removed. This suggests that the magnitude of the environmental trigger influences the heritability of the epigenetic response.

## Results

### Mouse Populations

**Figure 2:**
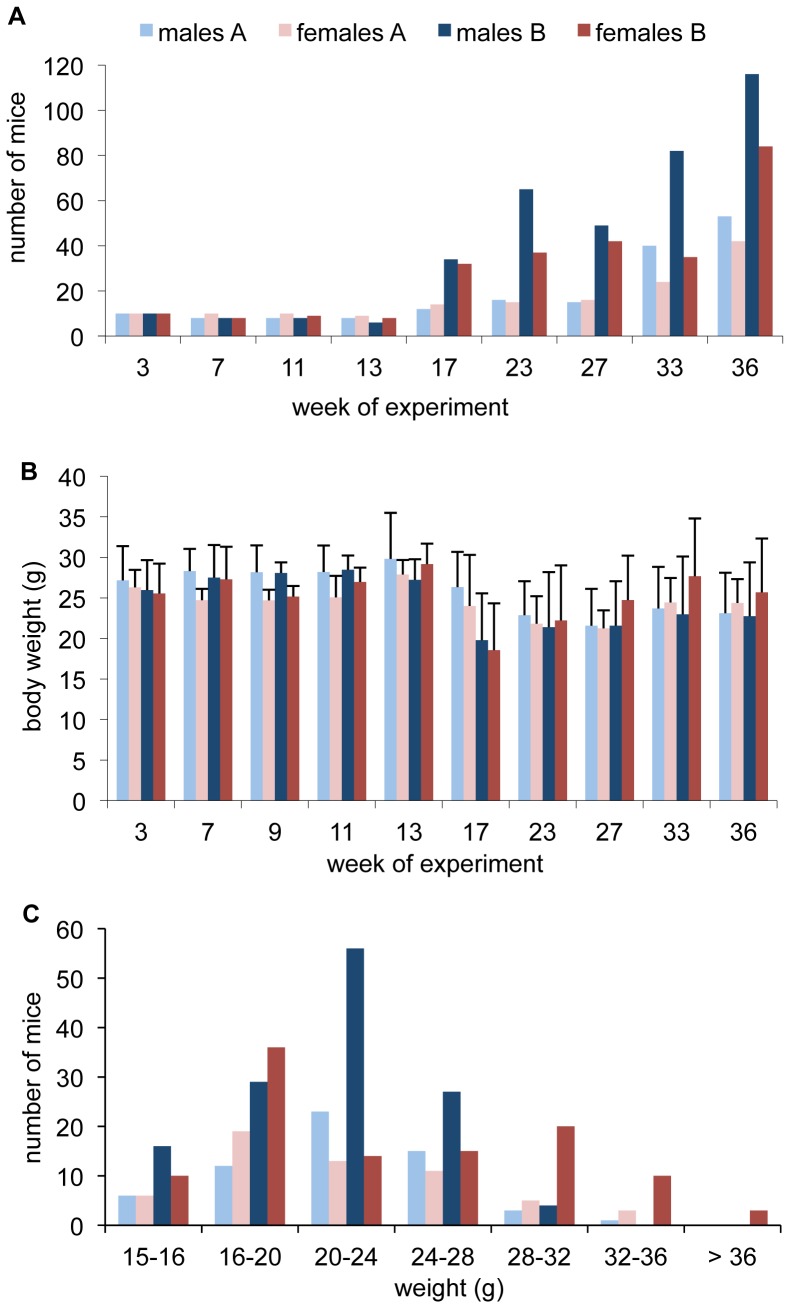
Mouse numbers and body weights during the course of the experiment. (A) Number of adult mice during the enclosure experiment. After week 13, the founder generation was removed from the experiment. Further reductions of mouse numbers were necessary in population B in week 25 (32 males, 18 females), week 28 (55 males, 25 females), and week 34 (46 males, 23 females). (B) Weights of adult mice are shown as means ± sd. The decrease in week 17 is due to the removal of the founder generation which led to an increase in the proportion of younger adult mice. This effect was larger in population B, as more pups had been born during the preceding weeks. (C) Weight distribution of adult mice at sampling time (week 36).

Once the first generation of mice born in the enclosures had reached adolescence, the number of adult mice increased steadily during the experiment (Figure 2A). In population A, this increase was greatest after week 33 and coincided with a distortion of the sex-ratio towards a higher number of adult males. Population B on the high-energy diet grew already faster after week 17, and by week 23 there were consistently more males than females (sex ratio 1.2–2.3). Since the founder animals were removed in week 13, this sudden increase in population size and number of litters (population B) represents the start of sexual activity of the first generation born in the enclosures. The faster growth of population B is probably an effect of the energy-enriched diet [48], which is designed by the manufacturer to enhance breeding success. A male-biased sex allocation has previously been described in a mouse population where sufficient but moderate food availability stimulated competition between females, and the reproductively successful dams produced more male offspring [49], [50].

The mean weights of adult mice (excluding pregnant females) did not significantly differ between the two populations throughout the experiment (Figure 2B). Moreover, the weight distribution was similar in both populations at sampling time (Figure 2C): most males weighed between 20–24 g and most females between 16–20 g. The second maximum of female weights around 30 g in population B comprises pregnant and lactating individuals.

### ChIP-Seq of H3K4me3 Enriched Wild-mouse DNA

**Figure 3:**
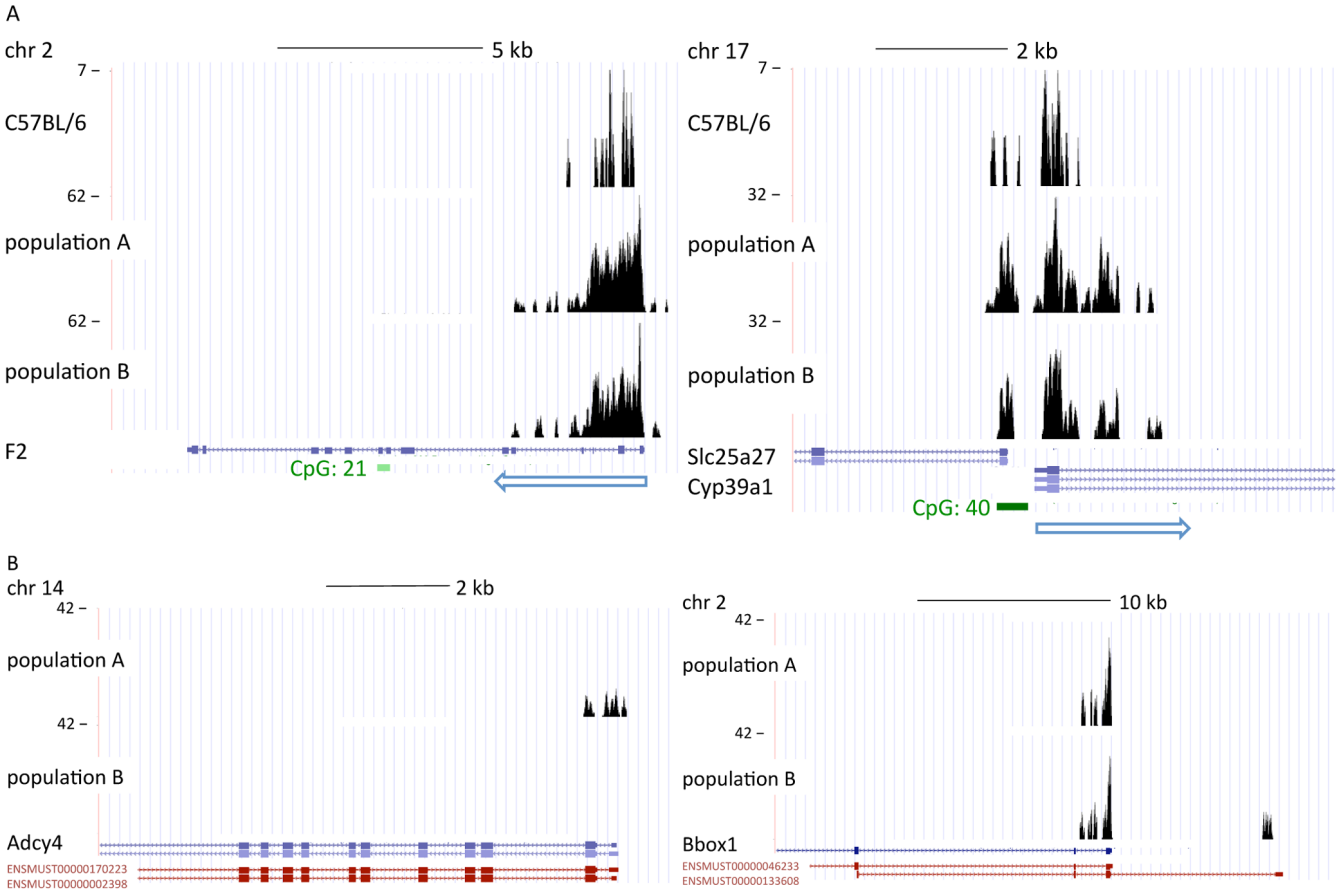
Examples of H3K4me3 markings at liver genes. Custom tracks are shown on the UCSC genome browser. (A) H3K4me3 peaks of the reference dataset by Robertson et al. [51] and experimental populations A and B for the coagulation factor prothrombine (F2) and the primary bile acid biosynthesis enzyme Cyp39a1 (24-hydroxycholesterol 7-alpha-hydroxylase). Blue arrows show the direction of transcription. (B) Examples of the largest exclusive differences between populations A and B. Peak sizes at the adenylate cyclase *Adcy4* (population A: 2,619 reads) and at the gamma-butyrobetaine hydroxylase *Bbox1* (population B, minor marked TSS: 3,013 reads) were much smaller that the peaks shown in (A).

From each of the populations A, B, A′, and B′, we sampled eight healthy males. Chromatin preparations for each individual were pooled within populations resulting in four ChIP-Seq libraries. The mapping of our ChIP-Seq reads onto the mouse reference genome (mm9) resulted in between 60% to over 70% uniquely mapped 36 bp reads (Table S1), which agrees well with previous ChIP-Seq experiments from inbred C57BL/6 mice (54%; [51]. H3K4me3 markings clustered within a window of 2,000 bp around TSS (Figure S1) with a gap indicating dismantling of nucleosomes at active TSS. Transcription end sites were not marked [42], [46]. The number of marked genes, the percentage of marking within functional gene sets, and the H3K4me3 pattern at typical liver loci (Figure 3A) agreed with the reference mouse dataset (Text S1). This confirms that our ChIP-Seq runs from wild-caught mice returned a reliable liver H3K4me3 profile.

### H3K4me3 Marking Differences

There were virtually no qualitative differences in H3K4me3 markings between populations A and B, as the few peaks present in one but absent in the other population were very small (Figure 3B, Text S1). Such exclusive peaks were often the result of a minor H3K4me3 mark at alternative TSS annotations, while the major mark showed no difference between the two populations (Figure 3B).

The overall H3K4me3 peak size distributions were very similar for all four populations (Table S2, Table S3, Figure S4). However, genome-wide correlations of peak sizes between population A and B were slightly lower than those of the offspring populations A′ and B′ at both TSS and at annotated CpG islands that were marked by H3K4me3 (Figure S5, Table S4). The difference at TSS was greater within the set of differentially expressed genes (Table S4, Figure S6). To further analyze the genome-wide peak size distributions between our experimental and offspring populations, we used a Mann-Whitney-Wilcoxon Test and permutation tests. In both tests, the medians of TSS peak sizes were significantly changed between populations A and B (*P* = 0.014 and *P* = 0.005, respectively) but not between populations A′ and B′ (*P* = 0.331 and *P* = 0.773, respectively). The variances were not different (Table S5). H3K4me3 markings at annotated CpG islands were unchanged for the AB comparison and the A′B′ comparison (Table S5, Text S1). As a control, we also performed the Mann-Whitney-Wilcoxon Test after reducing the TSS peak dataset of 33,940 annotated transcripts to one peak per gene by maximal difference scores A′B′ instead of AB. This resulted in 11,727 marked genes. Again, the experimental populations A and B were significantly different (*P = *0.028), but not the offspring A′ and B′ (*P = *0.281). We conclude that the two environments caused a reversible epigenetic response.

The measure we used to quantify differences between peak sizes, the difference score, is the product of absolute difference and fold change. This allows a comparison over the full range of the peak size distribution (Figure S3). The bulk of the difference scores clustered at TSS peak sizes between 6,000 and 20,000 aggregate sequence tag coverages (Figure S7A). The genome-wide distribution of difference scores AB (comparison between experimental populations) was shifted towards lower values in the comparison A′B′ (offspring bred under standard conditions; Figure S7B). This shift was highly significant as determined by the Mann-Whitney-Wilcoxon Test (*P*<2.2*10^−16^). Mean, skewness, and kurtosis of the difference scores AB were similarly moved towards lower values in scores A′B′ (Table S3). Thus, the difference scores showed plasticity in H3K4me3 markings in agreement with the comparison of peak size distributions. In order to assess how many genes contributed to H3K4me3 differences, we ranked all genes by their difference score AB, repeatedly omitted the most diverse genes, and re-calculated *P* values for the remaining dataset (Figure S8). Between 900 to 1,000 genes contributed to the H3K4me3 variation between populations A and B at a significance level of 0.05.

**Figure 4:**
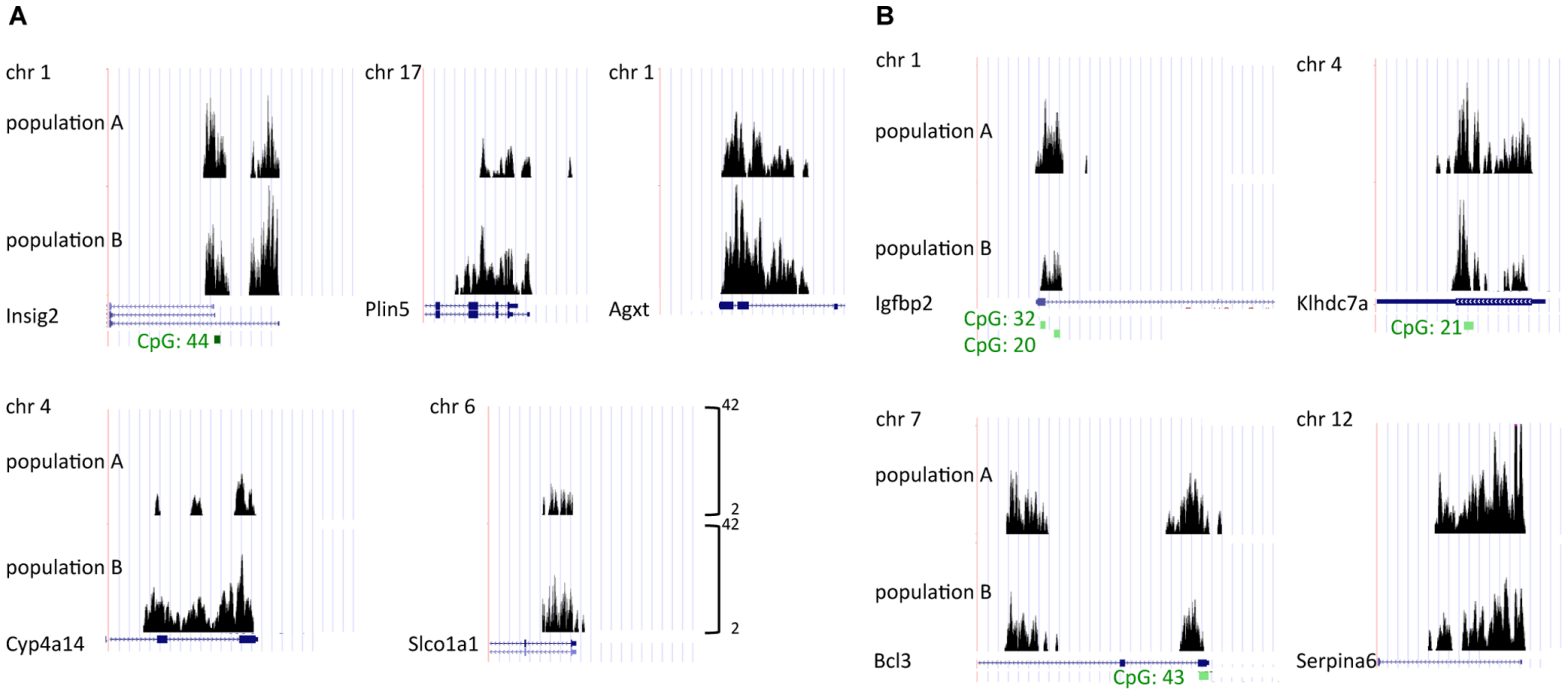
Examples of quantitative H3K4me3 differences between the experimental populations. (A) H3K4me3 peaks were upmarked in population B at cholesterol biosynthesis regulator *Insig2* (position chr1∶123,229,166), *Plin5* (intracellular lipid storage droplet protein), *Agxt* (glyoxylate detoxifier), *Cyp4a14* (an arachidonic acid monooxygenase), and *Slco1a1* (bile acid/organic anion transporter). (B) H3K4me3 marks were reduced in population B at the TSS of insulin-like growth factor binding protein 2 (*Igfbp2*), membrane protein *Klhdc7a*, the immune regulator *Bcl3* (position chr7∶20,408,104), and the blood glucocorticoid transport protein (alpha globulin) *Serpina6*.

Examples of H3K4me3 marking differences between the two experimental populations are shown as custom tracks (Figure 4). For genes *Insig2* and *Bcl3*, the marking differences were only found at one annotated TSS (ENSMUST00000071064 and ENSMUST00000065454, respectively). Altered H3K4me3 markings affected genes with or without CpG islands overlapping TSS (green annotation in Figure 4).

**Figure 5:**
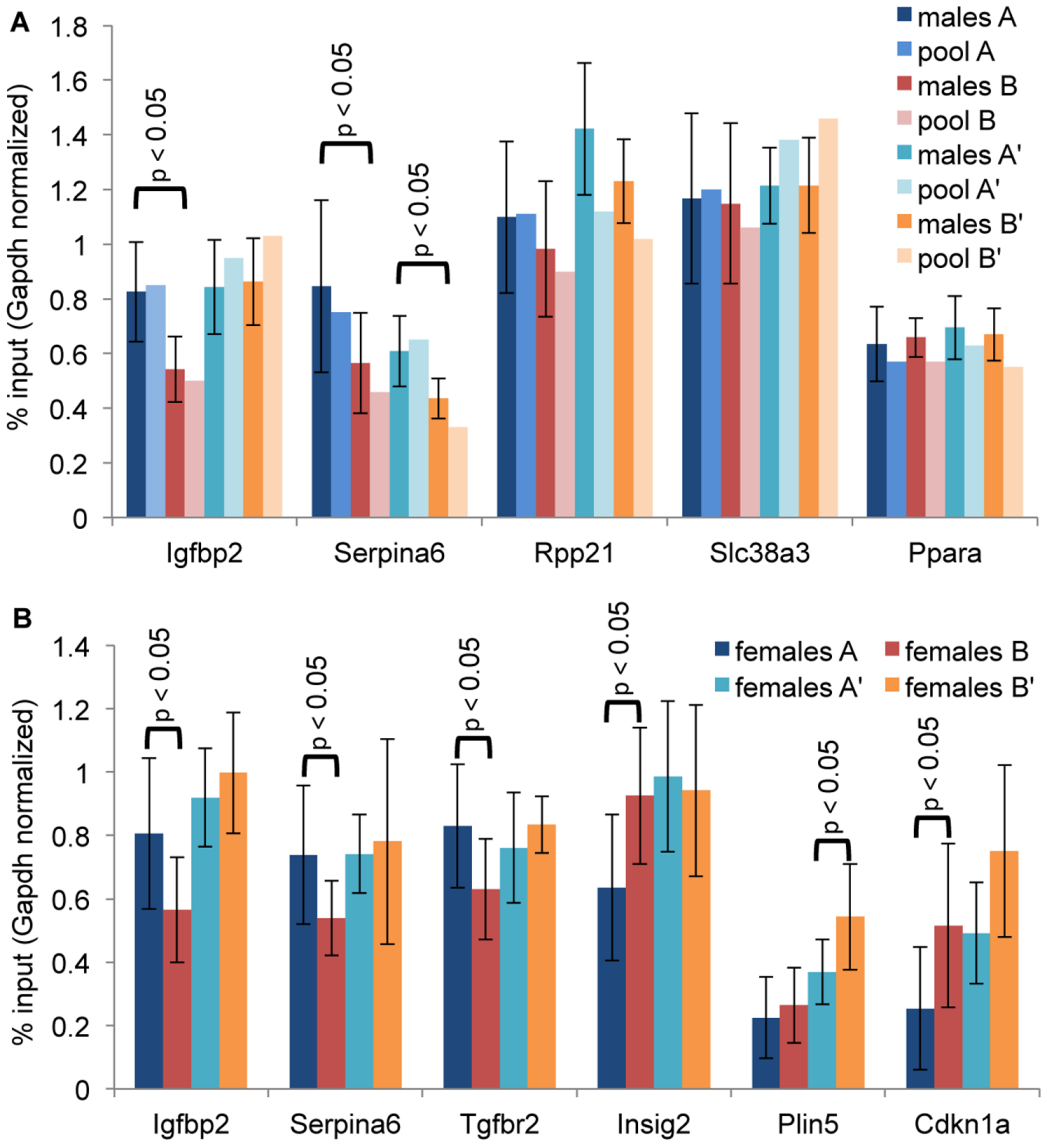
Quantification of H3K4me3 enrichment by qPCR. ChIP-DNA was prepared from liver tissues of (A) 8 young males, or (B) 6 to 12 females. Enrichment of selected genes was analyzed with respect to the input control (before IP) and normalized to *Gapdh*. Shown are means and standard deviation. Statistical significance was tested by a two-sided T-test.

To validate ChIP-Seq measurements by a second method and to include more animals for testing statistical significance of the H3K4me3 signal, we quantified enrichment of ChIP-DNA by qPCR. The qPCR approach has the limitation that the amplicon sizes of 80 to 160 bp do not fully reflect the 2 kb of ChIP-Seq peaks. Nevertheless, we found statistically significant differences of H3K4me3 markings between populations A and B at *Igfbp2* and *Serpina6* in males (Figure 5A) as well as in females (Figure 5B). ChIP-seq differences at loci *Tgfbr2, Insig2,* and *Cdkn1a* were confirmed in females (Figure 5B) and in young females at locus *Plin5* (*P* = 0.052, n = 6, not shown). The qPCR measurements of the pools that had been sequenced well agreed with individual preparations (Figure 5A). At loci *Rpp21* (difference score −15232), *Slc38a3* (difference score −5472) and *Ppara* (difference score 4588), the qPCR quantification agreed with the ChIP-Seq measurement but was not significant. Differences at most loci were not present in the offspring comparison. Significant exceptions were *Serpina6* in males (but not in females) and *Plin5* in females. Similarly, downmarking at *Rpp21* and upmarking at *Cdkn1a* were found to be stable in the offspring, but not at a significant level.

Comparisons of H3K4me3 enrichment between populations A and B were repeated using another histone marker that is strongly associated with active genes, the modification histone-3-lysine-27-acetylation (H3K27ac; [52]). The H3K27ac enrichment patterns agreed well with the H3K4me3 marks (Figure S9).

### Functional Analysis

Loci with difference score values AB greater than the 99%tile were selected for functional analysis, and the comparison to the A′B′ difference score was used to evaluate their plasticity (Table 1). For this analysis step, the computed peak quantification was verified manually over the exact H3K4me3 window (Text S1). Of the resulting 77 genes, 48 were upmarked and 29 were downmarked in population B (Table 1; see also full data table in File S1). Most of these differences were lost in the offspring populations.

**Table 1:** Functional clustering of genes with largest differences in H3K4me3 markings between experimental populations.

FunctionalClass		Function	Genes with differences betweenpopulations A and B	Difference observed inoffspring comparison^b^	genomic info and known polymorphisms^c^	
**B upmarked**				**B′ upmarked**		
Metabolism	lipid^a^		Acad11, Cyp2d22, Cyp2e1, Cyp4a14, Cyp4f14, Dbi,Es31, Etnk2, Plin5, Slco1a1	Cyp2e1 (44%)	RFLP (1)	
			Insig2	Insig2 (32%)	ENSMUST00000071064	
			Npas2	Npas2 (71%)	PCR (1)	
	carbohydrate		G6pc, Glo1, Nr0b2, Ppp1r3b, Ppp1r3c	Glo1 (27%)	within CNV region	
	amino acid		Agxt, Gnmt, Hpd			
	other		Fmo5, Mthfr			
Cell cycle	apoptosis; arrest		Cdkn1a, Ddit4, Dusp1, Gadd45g, Slc11a2			
	proliferation		Rad54l2	Rad54l2 (44%)		
Immune	defense response		Lbp, Uba5	Uba5 (29%)		
	inflammation		Cd97, Serpina3k, Serpina3m			
Transcription/translation	RNA binding		Pop4, Srrm4, Zfp36l1	Pop4 (89%)	within CNV region	
	DNA binding		Nfib, Npas2, Nr0b2			
Endoplasmatic reticulum	stress response		Herpud1, Mgst1	Herpud1 (52%)		
other	–		Btbd9, Slc25a25, Tmie	Btbd9 (60%)	within CNV region	
unknown	–		1600014C10Rik, 1810011O10Rik, AC124005.1,Dscr3, Pnkd	1600014C10Rik (>100%)	within CNV region	
			Anubl1	Anubl1 (94%)		
**B downmarked**				**B′ downmarked**		
Metabolism	lipid		Acacb			
	carbohydrate		Ppp1r3g			
Cell cycle	apoptosis; arrest		Fnip2, Igfbp2, Ypel3			
	proliferation; differentiation		Hjurp, Tnfaip2	Hjurp (>100%)		within CNV region
Immune	defense response		Bcl3, Bcl6,	Bcl3 (89%)		RFLP (3)
			H2_L	H2_L (27%)		RFLP (12)
	inflammation		Abcf1			
Transcription/translation	RNA binding		Cwc22, Elac1, Rpp21, SSU_rRNA_5	Cwc22 (32%)		within CNV region
	DNA binding		Arid1a, Cwc22, Dbp	Cwc22 (32%)		within CNV region
Signaltransduction	various		Fzd8, Ralgps2, Tgfbr2			
Hormones	regulation,biosynthesis		Dio1, Serpina6			
other	–		Spp2			
unknown	–		1110034G24Rik, AC132460.2, E030030l06Rik,Klhdc7a, Pcp4l1, Rnf186	Klhdc7a (92%)		

a including arachidonic acid metabolism.

b Genes where the H3K4me3 marking difference between the two experimental populations was also observed between the offspring populations. Numbers are the percentage difference score A′B′ of difference score AB.

c polymorphism report of www.informatics.jax.org. RFLP: restriction fragment length polymorphism.

The largest functional group upmarked in population B consisted of metabolic genes, which clustered into lipid and arachidonic acid metabolism (12 genes), carbohydrate metabolism (5 genes), and amino acid metabolism (3 genes). Only two metabolic genes were found to be downmarked. The numbers of genes upmarked and downmarked were similar for the categories cell cycle, immune function, and transcription/translation. Expression levels (see below and File S1) within this gene set were high for 71% of the genes found upmarked in population B (66% of downmarked genes), medium for 19% of the genes upmarked in B (10% of downmarked genes), and low for only 2% of the upmarked genes (14% of downmarked genes). This indicates that the majority of genes with a change in H3K4me3 marking comes from the set of highly expressed genes.

The analysis of gene set enrichment by KEGG or GO annotations at a significance level of 0.05 resulted in clusters of metabolic and immune functions (File S2). Enriched KEGG pathways were drug metabolism, arachidonic acid metabolism, and the insulin signaling pathway. Enriched GO categories of biological processes were cellular lipid metabolic process, regulation of carbohydrate metabolic process, and immune responses (leukocyte activation). When we submitted the 16 genes with at least partial transmission of H3K4me3 differences to the offspring populations as indicated in Table 1, none of the above pathways and categories were enriched (File S2). The only significant result was GO category “endoplasmatic reticulum membrane” including three genes (*Insig2*, *Cyp2e1*, and *Herpud1*) out of 173. Since this is a cellular component annotation, rather than biological function, and since there are no KEGG pathways shared by at least two of these genes, it is unlikely that this is a functionally driven transmission of epigenetic signals.

**Figure 6:**
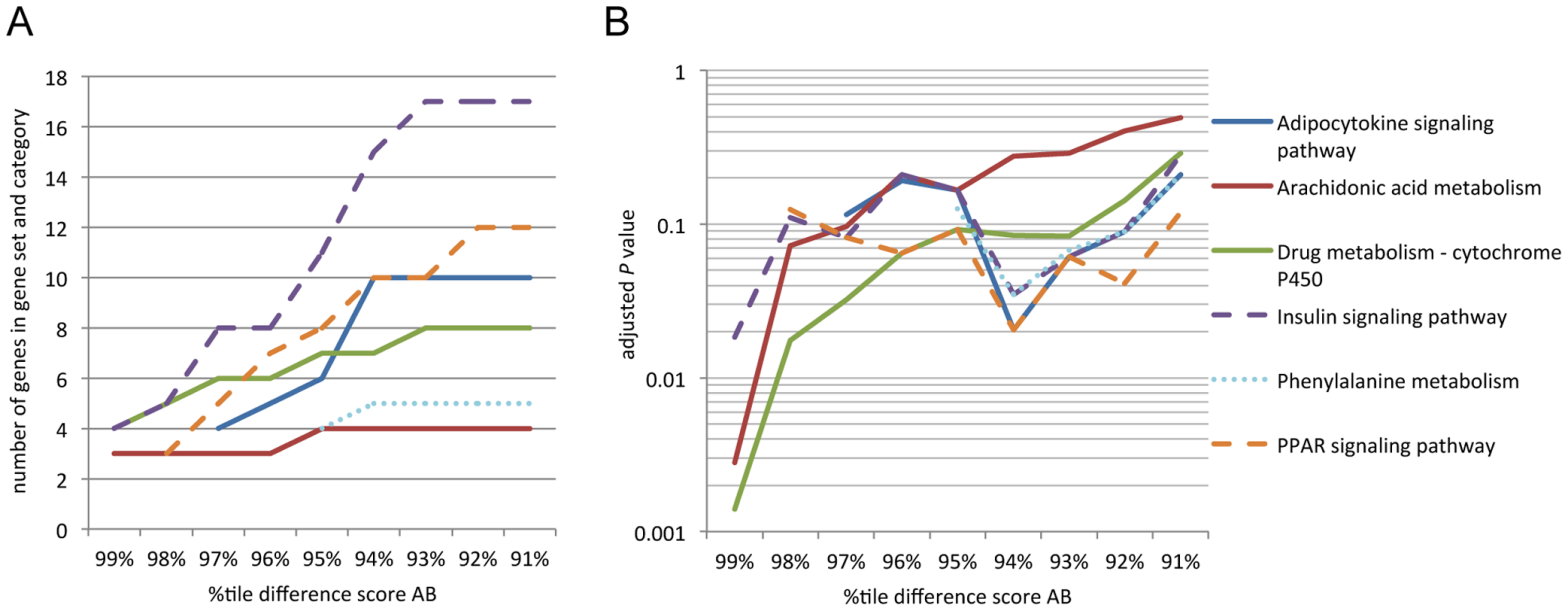
Functional enrichment analysis. Genes were ranked by difference scores AB and gene sets were selected along the top percentiles of the difference score distribution. Enrichment was analyzed against all genes that were marked in the four populations (11,070 loci). (A) Number of genes present in the gene set and in the category are shown for selected pathways. (B) Adjusted *P* values (Benjamini-Hochberg correction) tend to decrease with increasing numbers of genes submitted to the analysis.

In addition to large difference scores, modest shifts might contribute to gene regulation. We therefore also tested KEGG pathway enrichment of larger gene sets. The number of genes within enriched categories increased stepwise until reaching a plateau (Figure 6A). Adjusted *P* values decreased accordingly (Figure 6B). Enrichment of arachidonic acid metabolism and insulin signaling was already lost in the 98%tile gene set, but enrichment of cytochrome P450 drug metabolism stayed significant up to the 97%tile gene set. PPAR signaling and adipocytokine signaling, both involved in regulating lipid metabolism, were significantly enriched again at the 94%tile to 92%tile analysis levels as were, to a lesser degree, insulin signaling and phenylalanine metabolism.

### Epigenetic Differences on the Background of Wild-type Genomes

By far the largest difference in both H3K4me3 marking and expression was observed at *Cwc22*, a spliceosome factor involved in pre-mRNA splicing [53]. The background marking of a 150 kb region around this gene was massively increased (Figure S10A), which is probably due to the mapping of reads from several genetic copies to one annotated copy in the reference genome. Similarly, we found an increase in background reads at a region around the methylglyoxal detoxifier *Glo1* (glyoxalase 1; Figure S10B), a gene that is associated with anxiety in mice [54], [55]. A third suspected copy number variant spans a region containing genes *1600014CRik*, *Plekhf1*, and *Pop4*, all of which were differently marked between populations A and B (Figure S10C). Thus, the H3K4me3 differences of a handful of genes were the result of structural variations that were present in our wild mouse population. These differences were also observed in the offspring populations to various degrees (Table 1). When quantifying epigenetic marks in wild organisms, one needs to take into account that these are possibly under the influence of structural variation. For example, sequence polymorphisms such as restriction fragment length polymorphism (RFLP) and PCR polymorphism have been described for a few of the genes listed in Table 1. The H3K4me3 marking differences found at these loci could be caused by mutations.

It is not known to which extent single nucleotide polymorphisms (SNPs) influence the size of histone marks [56]. To estimate possible effects of the number of SNPs per locus on H3K4me3 marks, we compared Watterson’s theta of all 11,070 marked genes (mean theta/bp = 6.95×10^−3^) with that of the top 77 differently marked genes (mean theta/bp = 7.45×10^−3^). Theta values of these two gene sets differed significantly (*P* = 0.033, Wilcoxon rank sum test), but significance was lost at the 98%tile analysis level and beyond. Thus, highly polymorphic genes are over-represented among the set of most differently marked genes. A similar result was recently reported from human tissues where SNPs that are listed in the GWAS catalogue for human diseases and traits were significantly enriched within cell-type specific differentially DNA-methylated regions [57].

### Differential Expression

Since H3K4me3 characterizes open chromatin, we compared expression data from our sampled mice with the H3K4me3 signal. Of 14,779 genes expressed in populations A and B, only 216 were differentially expressed (adjusted *P*<0.05). Seven of these genes overlapped with the most differently marked genes. The probability of drawing seven or more of the 77 most differently marked genes when randomly sampling 216 out of the 11,070 marked genes is.
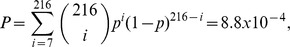
where *p* = 77/11,070. In other words, the differentially expressed genes are highly enriched for differently marked genes. The seven genes were *Btbd9*, *Cwc22*, *Glo1*, and *Hjurp* as genes from copy number loci, and the monooxygenase *Fmo5*, the DNA-response gene *Fnip2*, and the bile acid transporter *Slco1a1*. Only H3K4me3 differences within copy number loci were retained in the offspring populations (Figure S6 and Table 1).

### Stable H3K4me3 Patterns

Within the set of 77 most differently marked genes, the proportion of genes with stable H3K4me3 differences was 5.2% (difference score A′B′ at least 90% of score AB) and 2.8% when CNV loci were not counted (Table 1). At moderately changed loci (gene set of the 94%tile analysis), stable marking differences were reduced to 2.1% and 1.7%, respectively. Of the 14 genes with stable difference scores from the latter analysis (Table 2) only two genes had metabolic functions. The gene of the NADPH generating enzyme H6pd, which is involved in gluconeogenesis, was upmarked in populations B and B′. TSS marking occurred at two annotated TSS, and the higher marking was also the altered marking. *H6pd* transcription is upregulated in non-fasting, diabetic rats [58]. Downmarked in both populations B and B′ was the steroid hormone receptor *Pparg*, a regulator of lipid metabolism in adipocytes [59]. In addition, difference scores were stable at four zinc finger protein genes (Table 2).

**Table 2:** Patterns of inheritance from the gene set with the difference scores AB >94%tile where difference scores A′B′ were at least 90% of the AB value.

genesymbol	differencescore AB^a^	differencescore A′B′^a^	functional category	genomic info
**B and B**′ **upmarked**				
1600014C10Rik	27459	96341	ND	within CNV region
Anubl1	9484	8961	zinc finger protein	
Il1r1	6508	9781	innate immune response	
Slc45a3	6172	6656	prostate cancer associated protein; protein family H+/sugar transporter	
H6pd	5735	5813	glucose metabolic process	
Plekhf1	5121	6844	apoptotic process; lysosome	within CNV region
Cyp26a1	5034	5175	regulation of gene expression (cellular retinoid level)	
**B and B′ downmarked**				
Znrd1	−4460	−5275	transcription: zinc finger protein; DNA-directed RNA polymerase activity	
Xaf1	−5073	−5179	zinc finger protein; apoptotic process	
Zc3h7a	−5494	−22463	zinc finger protein	
Pparg	−5760	−5827	steroid hormone receptor activity; lipid metabolism	
Rsl1d1	−6637	−8759	structural constituent of ribosome	
Klhdc7a	−14652	−13494	ND; membrane protein	
Hjurp	−16068	−17086	cell cycle: nucleosome assembly	within CNV region

a difference scores calculated from exact peak windows.

## Discussion

Our genome-wide scan of differences in H3K4me3 marks induced by mild environmental fluctuations in wild-caught house mice demonstrates the adaptability of epigenetic regulation under near-natural conditions. Epigenetic variation underlies part of phenotypic variation [60] and is an important substrate for natural selection in the wild, especially if epigenetic settings respond to environmental change [33]–[35], [37]. Studies on human blood allow the assessment of epigenetic features in outbred genomes [7], [61] but are not amendable to easy experimental manipulation. However, in humans parental or grandparental availability of food is known to correlate with cardiovascular mortality risk in offspring [62] which illustrates the potential for environmental conditions to heritably affect the epigenome and hence evolutionary fitness.

Our H3K4me3 ChIP-Seq data from wild-caught house mice was in good qualitative agreement with published H3K4me3 data from the reference mouse strain C57BL/6 [51]. Similarly, H3K4me3 markings remained qualitatively unchanged between the experimental populations, i.e. there were no prominent novel or missing marks. It thus appears that the measured epigenetic setting is liver-specific and that a moderate environmental change does not eliminate or induce H3K4me3 peaks. This contrasts with disease processes, where epigenetic settings, especially DNA methylation states, switch in parallel to the loss of the tight regulation of specific cell functions during tumor development [63]–[66].

We noticed early that the H3K4me3 pattern seemed to be modulated quantitatively through differences in peak heights rather than qualitatively through peak presence or absence. To account for the quantitative differences, we analyzed our data in three steps: first, normalize the H3K4me3 peaks using a set of house-keeping genes, second, apply a suitable difference measure, and third, link the quantitative differences with biological function. We applied a biological peak normalization, because the standard technical normalization through division by the number of tags was sensitive to differences in the number of small non-TSS peaks. Starting from current protocols for the analysis of gene expression data, we used the peak sizes of nine house-keeping genes [67], but the number of tags contributing to all TSS peaks would have given similar results. For the second step we needed a suitable metric to compare ChIP-Seq peak sizes ranging over five orders of magnitude. We found that the product of the absolute difference and the fold change, which we call difference score, much reduced distortions at the extreme ends of the peak size distribution and transformed the bulk of the dataset such that maximal differences could be detected between peaks of all sizes.

H3K4me3 peak size distributions were altered between the two experimental populations kept in differing environments, but not between their offspring bred under standard conditions. We showed this through a battery of tests that included comparing higher moments of the genome-wide peak size distributions, correlation coefficients, and median comparisons with the non-parametric Wilcoxon Rank Sum Test and its Monte-Carlo equivalent. The detection of epigenetic differences between the two populations is an important result as it demonstrates not only the plasticity of epigenetic settings, but also that comparative epigenetic studies can be conducted on a background of natural genetic variation. Nevertheless, we had to make sure that chromatin rearrangements and CNVs were not the only cause of these differences. Visualization of the data as browser tracks helped us to further analyze extreme changes. For example, there were (very few) peaks that did not display the typically ragged peak shape, but were tight, extremely high and often embedded in regions with reduced mappability due to repetitive flanking sequences. Four such peaks were found at location chr2∶98,501,941–98,509,766 within a cluster of satellite regions. A similar pattern with extreme H3K4me3 markings is seen in unstimulated C2C12 myoblasts, a cell-line derived from the C3H mouse inbred line (see Histone Modifications by ChIP-Seq from ENCODE/Caltech in the UCSC Mouse Genome Browser).

Because the study of epigenomics in outbred organisms is still in its infancy, it is important to couple the search for numeric differences with an analysis of biological plausibility. We investigated the functions of the top 1% changed H3K4me3 marked genes using their KEGG and GO annotation (Table 1). In addition, we analyzed enrichment of gene sets within the top 9% of differently marked genes. We found the largest changes in genes involved in energy metabolism, especially in lipid metabolism. These genes tended to be upmarked rather than downmarked in the treatment population, which agreed with our expectation of an increased metabolic demand by a nutrient-richer chow. Similar observations have been reported from gene expression studies where a high-fat diet caused the up- or downregulation of a number of metabolic genes [24], [68]–[71]. As these studies were conducted with laboratory animals and diverse diets, we would not expect a full overlap of genes that respond to the changed environment, but there are important matches. For example, seven metabolic genes (*Acox1*, *Acsl1*, *Cyp39a1*, *Cyp4a14*, *Dgka*, *Hsd17b12*, *Scp2*) and two transcription factors (*Ppara*, *Srebf1*), which responded significantly to the metabolic demand of a high-fat diet [69], were differently marked in our study (gene set >94%tile). Similarly, metabolic genes *Acsl1*, *Ddc*, *G6pc*, *Pck1*, nuclear factors *Hnf4a*, *Nrbf2*, *Ppara*, *Srebf1*, immune and cell cycle genes *Irf1*, *Il1r1*, *Pcna*, and signal transduction genes *Gna11*, *Rgs2* were differentially expressed in mouse liver tissue after a high-fat diet [68] and differently marked in our mice. Our study was designed to avoid pathological changes; it is all the more interesting that similar gene sets responded to our relatively mild treatment compared to medical experiments with diets that contain up to 70% fat.

We found one metabolic gene that was not described in previous feeding studies but that was differently marked between the experimental populations and partially also between the offspring: *Insig2*. Insig proteins inhibit fatty acid biosynthesis in a cholesterol-dependent fashion by blocking the processing of sterol regulatory element-binding proteins [72]–[74]. There was no statistically significant differential expression of *Insig2* due to a high within-group variation in our mice. However, the gene was highly expressed (log2 expression >13) in five mice out of the eight that were chosen to represent population B, but only in one mouse of population A (not shown). An upregulation of the Insig pathway would downregulate adipogenesis and block lipid synthesis, a response that might be expected under a fat-rich diet. However, the marking difference was partially retained in the offspring. There are two 5′ UTR sequence variants known in mice within the H3K4me3 marked *Insig2* TSS region. It is not known whether such sequence differences could influence an epigenetic signal and thus whether we observed epigenetic or genetic inheritance at this locus.

Any epigenetic study using outbred organisms runs into the problem of explaining epigenetic variation in the presence of genetic variation. To sequence candidate genes in wild populations will not necessarily explain epigenetic differences, because genomic variation at other loci, for example at a distant enhancer, could cause a specific H3K4me3 setting. To address the impact of SNP profiles on epigenetic settings would be an important step to understand possible epigenetic inheritance, especially in the absence, so far, of a molecular mechanism of epigenetic inheritance through the germ line. From the offspring of both populations that were raised under standard conditions, we learn that most of the subtle differences that pointed to changes in metabolic pathways were reversible. Structural changes such as CNVs were stable and led to epigenetic variation in the next generation. The interpretation of human ENCODE datasets will help to assess the influence of genetic variation on epigenetic modifications [75]. But to quantify responses to an environmental challenge in a social organism requires experimental manipulation of the type we have carried out.

Epigenetic inheritance in vertebrates has so far been demonstrated almost exclusively in toxic or pathological environments. Even the well-studied response of DNA methylation at the *Agouti* locus to a methyl-donor rich diet is based on a transposon insertion that triggers expression of a faulty transcript, i.e. on a mutation. The ongoing debate about the heritability of the pseudoagouti phenotype shows how difficult it is to disentangle germ-line epigenetic settings from environmentally triggered responses [76], [77]. The epigenetic silencing of potentially mutagenic transposons and the epigenetic adjustments to potentially lethal environments such as famine, high-fat diets, toxins, and psychological stress, suggest that inherited epigenetic changes are the drastic responses to an adverse environment. In contrast, the epigenetic plasticity we have observed might be the healthy, short-term adaptation that underlies fit phenotypes.

## Materials and Methods

### Ethics Statement

This study was carried out in strict accordance with German animal welfare legislation. The house mice used in our study (*Mus musculus domesticus*) were progenies of individuals captured in an area of a radius of 20 km around GPS location latitude: 50.71574, longitude: 6.916503 in 2007 [78]. House mice are not an endangered or protected species and there was no requirement for permission to catch mice in Germany at that time. Mice were captured in barns on private land with the oral permissions of landowners. Live traps provided food and shelter and were set at moderate temperatures by experienced personnel. Mice were handled throughout according to FELASA guidelines and German animal welfare law (Tierschutzgesetz §11). They have since been bred under a rotating outbreeding scheme [78] with permission from the Veterinäramt Kreis Plön (permit number 1401-144/PLÖ-004697). Animal work was registered under V312-72241.123-34 (97-8/07) and approved by the ethics committee of the Schleswig-Holstein State Ministry for Agriculture, Environment and Rural Areas.

### Treatment of Mice

The experimental populations started with mouse densities of approx. 1 mouse/m^2^ during the first weeks. Each enclosure contained 10 nest boxes, partitioning boards, and short plastic tubes that could be used as hiding places. Food and water were amply available, as well as nesting material. The feed was purchased from Altromin Spezialfutter GmbH & Co. KG, Lage, Germany. The standard diet (Altromin 1320) contained 2844 kcal/kg metabolizable energy (24% from protein, 11% from fat, and 65% from carbohydrates), the high-energy diet (Altromin 1410) contained 3154 kcal/kg metabolizable energy (27% from protein, 22% from fat, and 51% from carbohydrates). The light cycles were 12 h:12 h day−/night-time for population A (standard) and 18 h:6 h day−/night-time for population B (treatment).

### Mouse Monitoring

Enclosures were briefly inspected every day and dead animals were removed. Every second week, nests were examined, litters were counted, and as soon as the ears of pups lost their initial fleshiness (at an age of 2–3 weeks), ear punches were taken for DNA extraction. The location of the ear punches distinguished founder animals from animals born in the enclosures. Every four to six weeks, all mice were captured in order to monitor litter size, sex of mature mice, weight, fur condition and general health status of all mice. Any mice looking ill were removed from the experiment. After week 13, the founder animals were removed from the enclosures to ensure that only animals that were born in the experiment were left to multiply over the remaining time. Due to the rapid breeding in the treatment room, mouse numbers were reduced after weeks 25, 28, and 34 by randomly selecting a given percentage of mice from each nest and of those captured outside the nest boxes at that time point. Age of young to near full-grown mice was estimated using population-specific growth curves from our breeding facility.

After week 36, all mice were captured and counted. To avoid differences between females in various fertility states, we performed ChIP-Seq only on males. From each enclosure, eight young, healthy males (body weight 16–19 g, aged 4.5 to 6 weeks) were chosen from different nests as population representatives. They were killed by CO_2_ asphyxiation and cervical dislocation. Liver samples were snap frozen in liquid nitrogen, and stored at −80°. At the same time, up to three young mice (body weight 14–15 g) were selected from each nest and from the roaming mice. They were transferred to cages, kept under standard conditions with respect to diet and light cycle for 3 months, and were then bred among their experimental group. Eight healthy males (F_n+1_) from various breeding pairs were selected as representatives of population A′ and B′ (Figure 1).

### Chromatin Preparation, Immunoprecipitation and Illumina Sequencing

To obtain ChIP-Seq data, we followed standard procedures (Text S1). We used 100 to 120 mg of frozen mouse liver for the chromatin preparation. For ChIP-Seq, chromatin of eight animals per population was pooled containing 2 µg of DNA per animal. H3K4me3 was immunoprecipitated from each population pool using 10 µl of trimethyl-histone H3 (Lys4) rabbit monoclonal antibody (#9751, Cell Signaling). Quantitative PCR (qPCR) measurements were performed from enriched DNA of individual samples, starting with 2 µg of chromatin and using 2 µl of the anti-H3K4me3 antibody, 2 µl of the H3K27ac-antibody (#8173, Cell Signaling), or 0.5 µl of anti-rabbit IgG control antibody (#2729, Cell Signaling). High-throughput sequencing was performed on an Illumina Genome Analyzer IIx with 36 base pair (bp) read length. The number of sequencing reads, mapping success, and normalization factors are summarized in Table S1. Mapped reads were uploaded as custom tracks into the UCSC Genome Browser (version mm9) for visualization [79].

### Peak Calling and Quantification of Peak Sizes

A 2,000 bp window (TSS ±1,000 bp) at annotated transcripts of the ENSEMBL mouse database (assembly m37) was chosen to define TSS peaks (Figure S1). Peak sizes were calculated by summing the number of reads that mapped within this 2000 window and these aggregate coverages were stored in a relational database for further analysis. Exclusive peaks are H3K4me3 markings that were only present in either population A or population B. Peaks were normalized by the peak sizes of nine housekeeping genes [67] (Figure S2). For genes of interest, exact peak sizes were also calculated over a window determined by the precise peak width as seen in the custom browser tracks. For the analysis of CpG islands marked by H3K4me3, positions of annotated CpG islands were retrieved from table cpgIslandExt of the mm9 database (16,026 CpG islands) and mapped reads were summed within the corresponding window. CpG peaks were normalized by the sum of all reads mapping to CpG islands.

### Difference Score

We used the product between absolute difference and fold change as difference score (Figure S3). This calculation reduced the overestimation of differences from small peaks and the underestimation of differences from large peaks by a fold change calculation as well as the overestimation of differences from large peaks and the underestimation of differences from small peaks by an absolute difference calculation. For A<B, the difference score was (B–A)*(B/A) and had a positive value; for A>B, the difference score was (B–A)*(A/B) and had a negative value. For plotting log10 difference scores, absolute values were used.

### Merging Annotated Transcripts to One Gene

Our aim was to identify the most differently marked genes between populations A and B. We therefore reduced the 33,940 marked TSS to 11,995 marked genes by selecting for each gene the peak location where the window TSS±1000 bp resulted in the largest absolute value of the difference score. Very small peaks as defined by the lower whisker of a boxplot (outlier = quartile1–1.5*interquartile range) were discarded to minimize the risk that small markings distort the difference analysis. This resulted in a final set of 11,070 marked genes.

### Gene Set Enrichment Analysis

Gene set enrichment analysis was performed using the Web tool WebGestalt [80], [81]. Groups of genes were compared to the set of marked 11,070 genes rather than to all known genes. This restriction leads to more conservative enrichment criteria (higher *P* values) compared to the corresponding analysis against the genome. We performed the analysis as a hypergeometric test using the Benjamini-Hochberg correction for multiple testing and set a minimum of 3 genes per category to be required for enrichment.

### Quantitative PCR

H3K4me3 peaks from ChIP-Seq were validated by quantitative PCR (qPCR). In addition, females were tested. Measurements were performed in triplicates on 96 well plates in a volume of 10 µl per well consisting of 1 µl of purified ChIP-DNA, 5 µl of Fast Sybr Green Master Mix (#4385612, Applied Biosystems, Foster City, CA), 2 µl of primers and 3 µl of pure water (HPLC grade). The PCR reaction program was run according to the protocol of the SimpleChIP Enzymatic Chromatin IP Kit (Cell Signaling). Primer sequences are listed in Table S6.

### Expression Data

RNA was extracted from 20 mg of frozen tissue (−80°) using the PureLinc RNA Mini Kit (Life Technologies) with DNAse treatment. Quality assessment of the RNA preparation was performed using the RNA 6000 Nano Kit (Agilent Technologies, Santa Clara, CA) on an Agilent 2100 Bioanalyzer. Total RNA was labeled by cyanine 3-CTP using the one-color Quick Amp Labeling Kit (Agilent) and quantified using the Whole Mouse Genome Microarray Kit, 4x44K, (#G4122F, Agilent). Microarray slides were scanned on an Agilent G2565CA High-Resolution Microarray Scanner. Data were analyzed in R using the LIMMA package [82]. *P* values were adjusted by the Benjamini-Hochberg correction and differential expression was judged at the 0.05 significance level. For the assessment of high, medium, or low expression, the set of expressed genes was divided into three clusters using the expression level quantiles 66.7% and 33.3%. Each cluster was joined with the set of 11,070 H3K4me3 marked genes which resulted in 5,653 H3K4me3 marked genes with high expression, 4,441 with medium expression, and 2,772 with low expression.

### Statistical Analysis

Statistics of peak size distributions were calculated in R. Spearman’s rank correlation rho was used to describe the strength of association between the peak sizes of two populations. The Mann-Whitney-Wilcoxon Test (Wilcoxon rank sum test with continuity correction) was chosen as non-parametric test of independent data to compare peak size distributions. Monte Carlo simulations were programmed to assess the difference in median values and variances of the peak size distributions between two populations and were run with 100,000 itinerations.

Watterson’s theta [83] was calculated from 17 sequenced inbred mouse strains using SNP data published by the Sanger Center [84]. The table current_snps was accessed on 21/11/2012. Theta per bp was calculated over the length of each gene plus 2,000 bp upstream and downstream for all 11,070 marked genes.

### Data Repository

http://guanine.evolbio.mpg.de/~aboersch/dataMouseFeed/.

## Supporting Information

Figure S1 **Overlay plot of H3K4me3 coverages at transcription start sites (TSS) and transcription end sites (TES).** Peaks were selected within a window of 2500 bp downstream and upstream of mm9 annotated TSS or TES points. Coverages within this window were summed up and only peaks larger than 3,600 were included in the overlay plot (10,471 genes in sample A and 9,581 genes in sample B). To calculate the relative coverage, coverages per base pair with respect to its position within the TSS or TES window were summed up and were divided by the sum of the selected peak coverages.
(PDF)

Figure S2 **Normalization of H3K4me3 peak sizes by nine house-keeping genes.** (A) The comparison of custom browser tracks of population A and B′ at gene locus *Gapdh* demonstrates the presence of unspecific small read clusters in population B′ that were not present in population A. (B) Correlation of peak sizes of nine housekeeping genes before and after normalization: hydroxymethyl-bilane synthase (*Hmbs*), TATA box binding protein (*Tbp*), phospholipase A2 (*YWHAZ*), succinate dehydrogenase complex, subunit A (*Sdha*), beta actin (*Actb*), glyceraldehyde-3-phosphate dehydrogenase (*Gapdh*), ribosomal protein L13a (*Rpl13a*), beta-chain of major histocompatibility complex class I molecules (*B2m*), and ubiquitin C (*Ubc*).
(PDF)

Figure S3 **Dependence of various difference measures on peak size.** Three difference measures were calculated between population A and B at 11,995 genes. In this Figure, absolute difference was calculated as A–B for A>B and B–A for A<B. Fold change was calculated as A/B for A>B and B/A for A<B. The difference score is the product between difference and fold change.
(PDF)

Figure S4 **H3K4me3 peak size distributions of all four datasets.** (A) Peaks at TSS of 11,070 genes. (B) H3K4me3 markings overlapping CpG islands. 8,954 (56%) of 16,026 annotated CpG islands (mm9) were marked in our liver samples.
(PDF)

Figure S5 **Comparison of H3K4me3 peak sizes between experimental populations and their offspring.** Correlation of peak sizes from populations A and B or A′ and B′ at either the TSS (11,995 loci) or annotated and marked CpG islands (8,954 locations). Correlation coefficients are given as *r* (insert). Plot axes are limited at 60,000.
(PDF)

Figure S6 **Expression and H3K4me3 markings in experimental populations and their offspring.** Of 216 genes differentially expressed between populations A and B, 159 were also marked by H3K4me3. Plots are drawn with limited axes. Correlation of H3K4me3 markings between the experimental populations (black) and the offspring populations (red). The seven genes named in the plot are also among the 77 most differently marked genes.
(PDF)

Figure S7 **Difference scores.** (A) Difference score as function of H3K4me3 peak sizes. Data values at 11,995 loci (comparisons between populations A and B) are clustered in hexbins which range from 1 to 64 (increase in greyness). Box-whisker plots show median, quantiles Q1, Q3, and outliers. (B) Histograms of difference scores of the comparison between control and treatment (AB) and between the offspring populations (A′B′). The 11,070 data points were selected by omitting minimal peaks as defined by the lower whisker of the peak size distribution boxplot from population A, see (A).
(PDF)

Figure S8 **Mann-Whitney-Wilcoxon Test of reduced datasets after repeated omission of the most different loci.** H3K4me3 marked genes were ranked by the absolute value of the difference score AB and genes with the largest scores were consecutively omitted from the gene pool. *P* values of the original and the reduced gene pool datasets were obtained by a Mann-Whitney-Wilcoxon Test. Black dots show the comparisons between populations A and B, red dots between A′ and B′. The horizontal line is drawn at the *P* value of 0.05.
(PDF)

Figure S9 **qPCR measurements of H3K4me3 and H3K27ac enriched ChIP-DNA.** Individuals were eight young males from populations A and B, respectively, and shown are means and standard deviation. The *Gapdh* markings were lower in the H3K27ac ChIP-DNA compared to H3K4me3; therefore the normalized values appear to be larger.
(PDF)

Figure S10 **Copy number variant regions detected in our wild mouse population.** H3K4me3 tracks with a local increase in background reads indicate copy number variant loci. (A) The background of the chromosomal region chr2∶77,707,909–77,866,330 around spliceosome factor *Cwc22* was massively increased in population A. (B) The region chr17∶30,590,650–31,061,925 containing the genes *Btbd9* and *Glo1*, both upmarked by H3K4me3 in population B, is a copy number variant. Shown are tracks from population B′ as the increase in background reads was most noticeable here. (C) The three genes *1600014CRik*, *Plekhf1*, and *Pop4* were upmarked in population B and the increase in background (chr7∶38,959,201–39,058,174), again best demonstrated in population B′, points to a copy number variant region. The difference scores AB of genes *1600014CRik* and *Pop4* were higher than the 99%tile of the difference score distribution.
(PDF)

Table S1 **Sequencing, mapping, and normalization factors for the experimental populations A and B and the respective offspring populations A′ and B′.**
(PDF)

Table S2 **Peak size distribution of the normalized dataset.**
(PDF)

Table S3 **Four moments of data distributions.**
(PDF)

Table S4 **Spearman’s rank correlation rho of H3K4me3 marking comparisons between the experimental populations (AB) and the offspring (A′B′).**
(PDF)

Table S5 ***P*** **values of peak size distributions from comparisons of population A with B and A**′ **with B**′**.**
(PDF)

Table S6 **Primer sequences for qPCR measurements.**
(PDF)

Text S1 **Supporting Methods and Results.**
(PDF)

File S1 **Genes with the largest H3K4me3 differences between populations A and B.**
(PDF)

File S2 **KEGG and GO enrichment analysis.**
(PDF)
