# Supplementary figures and images for: Genome-Wide Quantitative Analysis of Histone H3 Lysine 4 Trimethylation in Wild House Mouse Liver: Environmental Change Causes Epigenetic Plasticity
Source: PLoS One. 2014 May 21;9(5):e97568. doi: 10.1371/journal.pone.0097568 (PMC4029994; doi:10.1371/journal.pone.0097568)

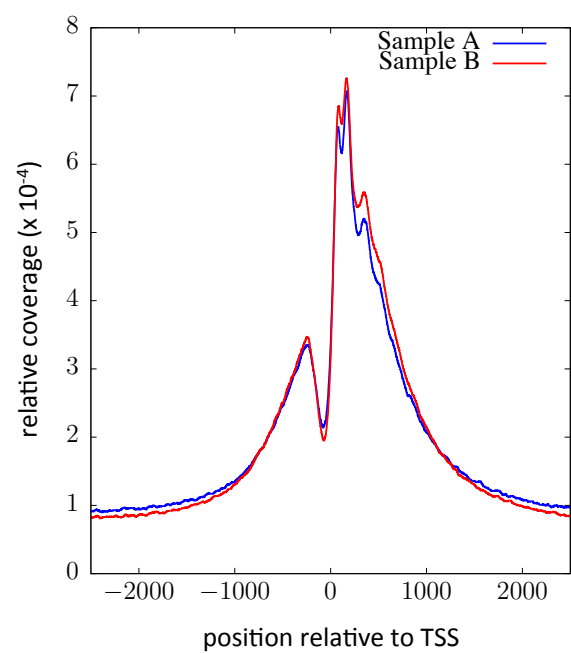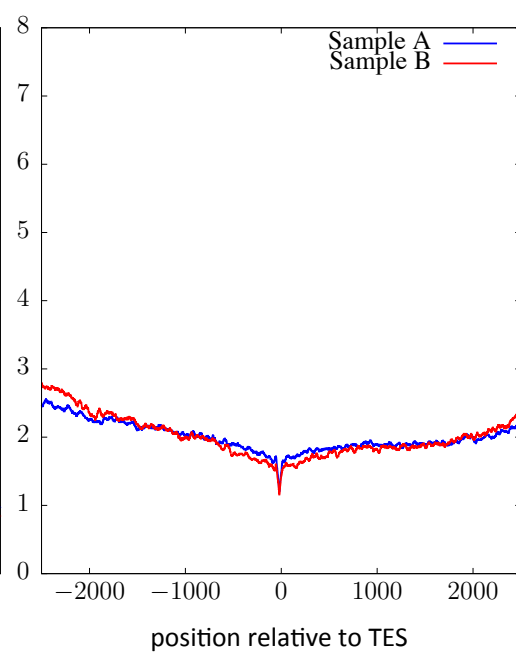

Supplement: Figure S1 — Overlay plot of H3K4me3 coverages at transcription start sites (TSS) and transcription end sites (TES). Peaks were selected within a window of 2500 bp downstream and upstream of mm9 annotated TSS or TES points. Coverages within this window were summed up and only peaks larger than 3,600 were included in the overlay plot (10,471 genes in sample A and 9,581 genes in sample B). To calculate the relative coverage, coverages per base pair with respect to its position within the TSS or TES window were summed up and were divided by the sum of the selected peak coverages. (PDF) [file pone.0097568.s001.pdf]

A

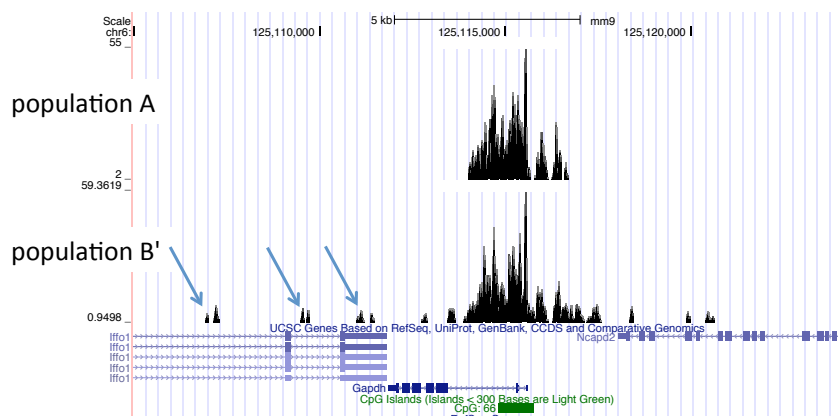

B

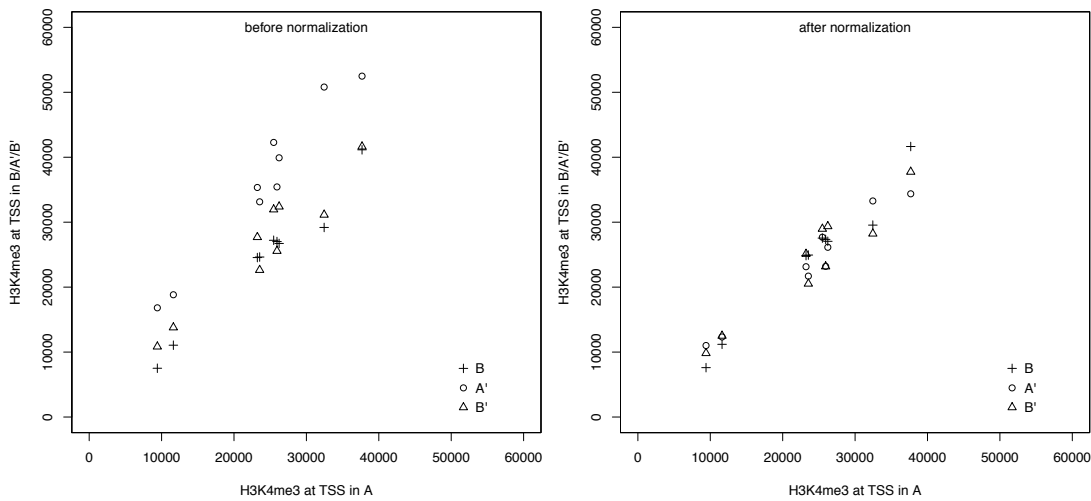

Supplement: Figure S2 — Normalization of H3K4me3 peak sizes by nine house-keeping genes. (A) The comparison of custom browser tracks of population A and B′ at gene locus Gapdh demonstrates the presence of unspecific small read clusters in population B′ that were not present in population A. (B) Correlation of peak sizes of nine housekeeping genes before and after normalization: hydroxymethyl-bilane synthase (Hmbs), TATA box binding protein (Tbp), phospholipase A2 (YWHAZ), succinate dehydrogenase complex, subunit A (Sdha), beta actin (Actb), glyceraldehyde-3-phosphate dehydrogenase (Gapdh), ribosomal protein L13a (Rpl13a), beta-chain of major histocompatibility complex class I molecules (B2m), and ubiquitin C (Ubc). (PDF) [file pone.0097568.s002.pdf]

**A**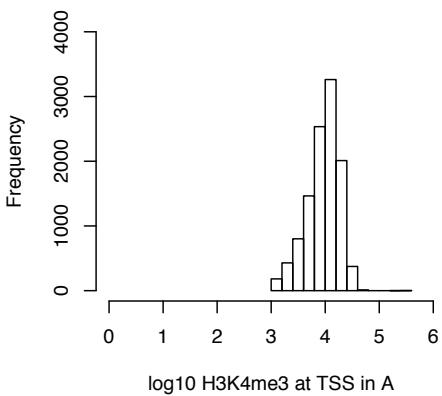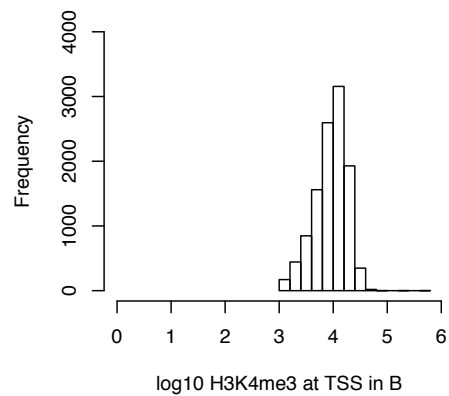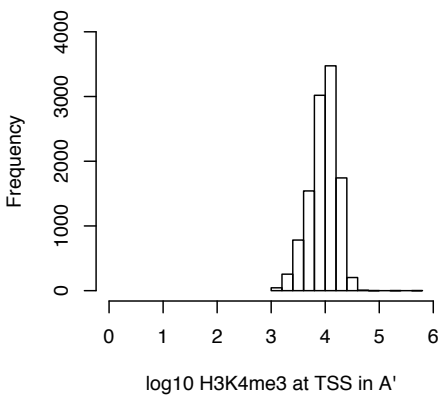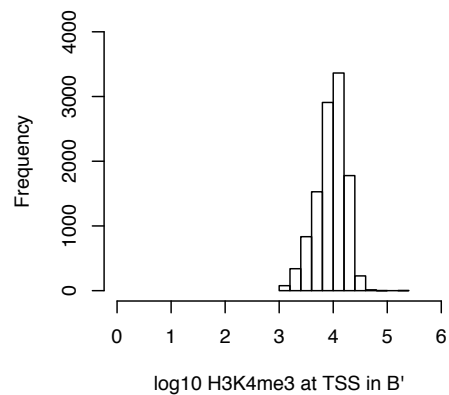**B**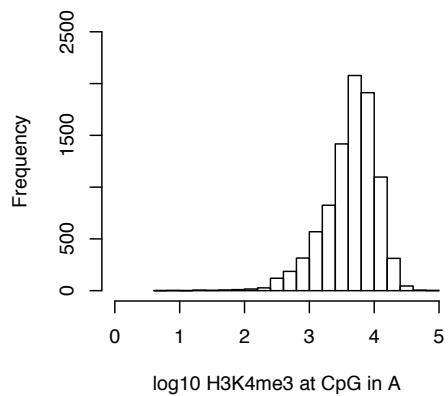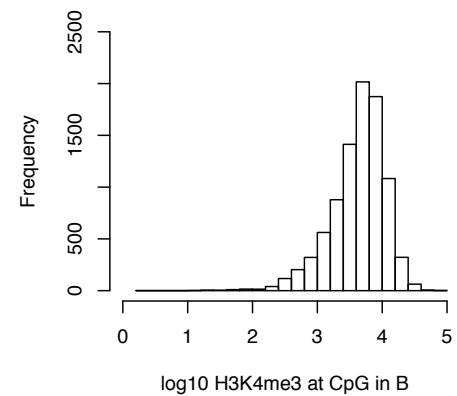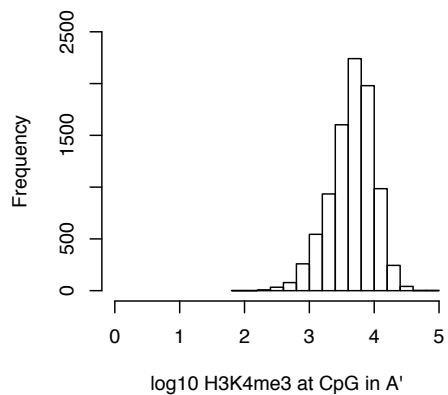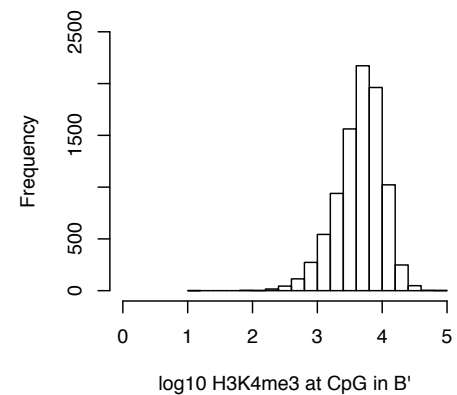

Supplement: Figure S4 — H3K4me3 peak size distributions of all four datasets. (A) Peaks at TSS of 11,070 genes. (B) H3K4me3 markings overlapping CpG islands. 8,954 (56%) of 16,026 annotated CpG islands (mm9) were marked in our liver samples. (PDF) [file pone.0097568.s004.pdf]

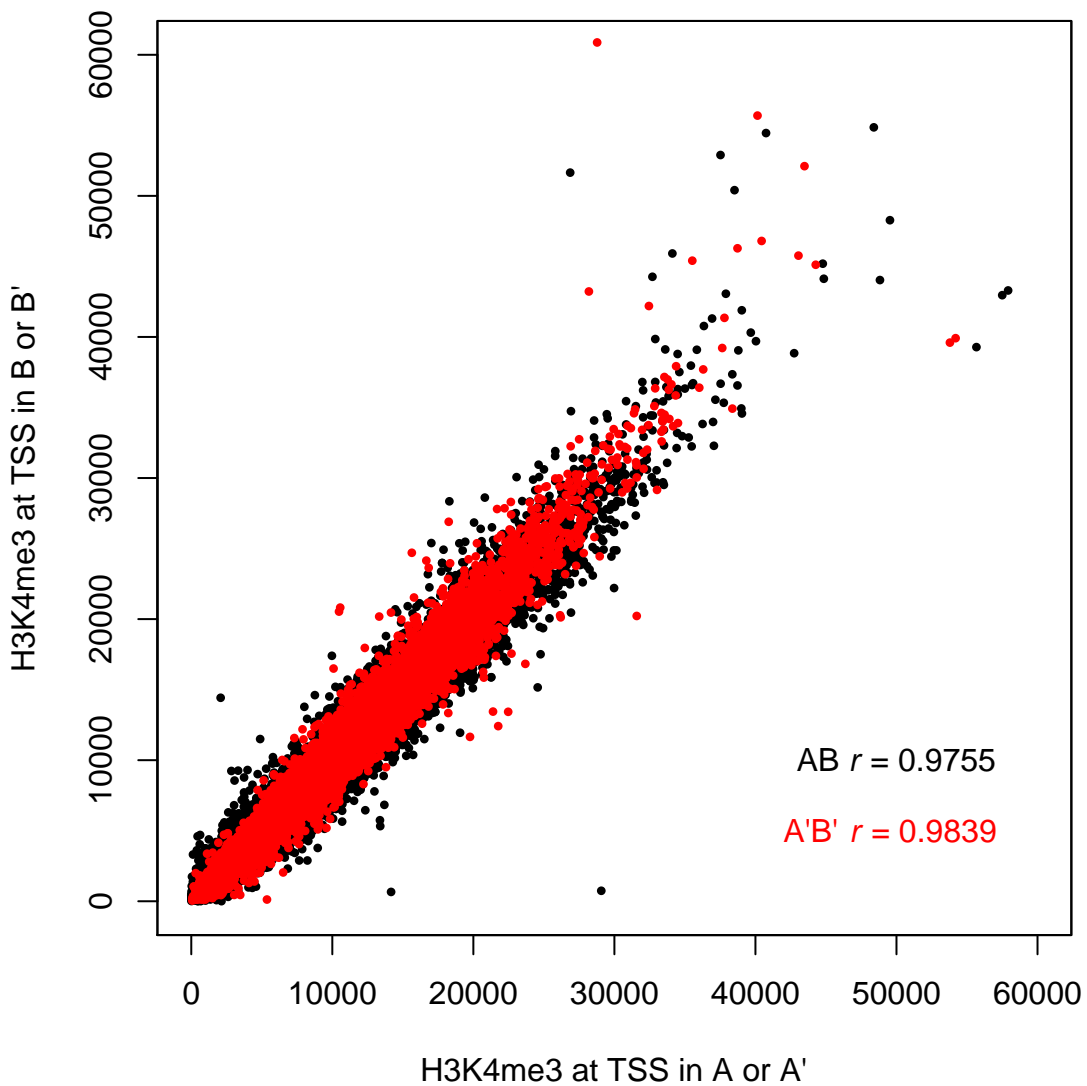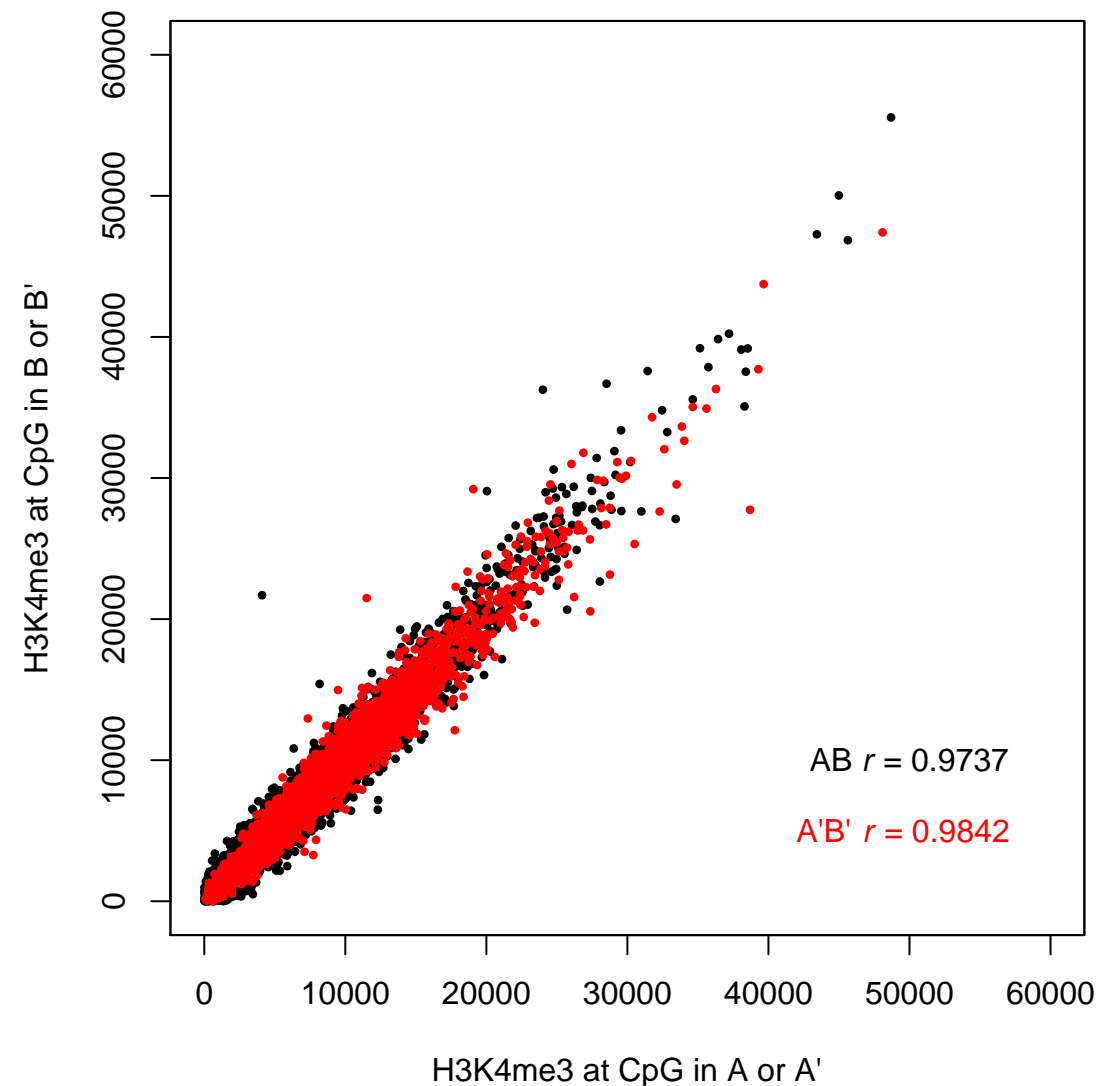

Supplement: Figure S5 — Comparison of H3K4me3 peak sizes between experimental populations and their offspring. Correlation of peak sizes from populations A and B or A′ and B′ at either the TSS (11,995 loci) or annotated and marked CpG islands (8,954 locations). Correlation coefficients are given as r (insert). Plot axes are limited at 60,000. (PDF) [file pone.0097568.s005.pdf]

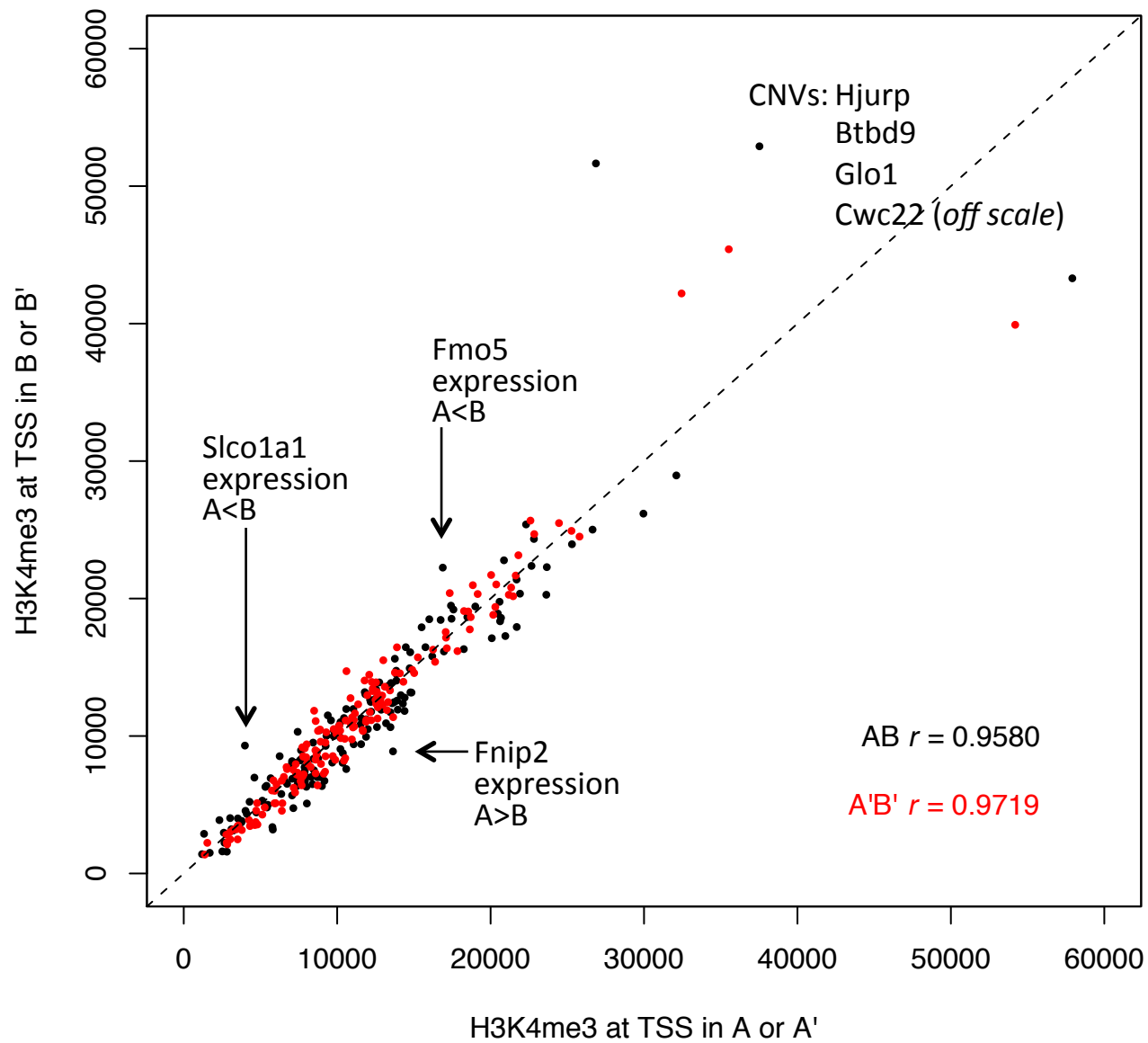

Supplement: Figure S6 — Expression and H3K4me3 markings in experimental populations and their offspring. Of 216 genes differentially expressed between populations A and B, 159 were also marked by H3K4me3. Plots are drawn with limited axes. Correlation of H3K4me3 markings between the experimental populations (black) and the offspring populations (red). The seven genes named in the plot are also among the 77 most differently marked genes. (PDF) [file pone.0097568.s006.pdf]

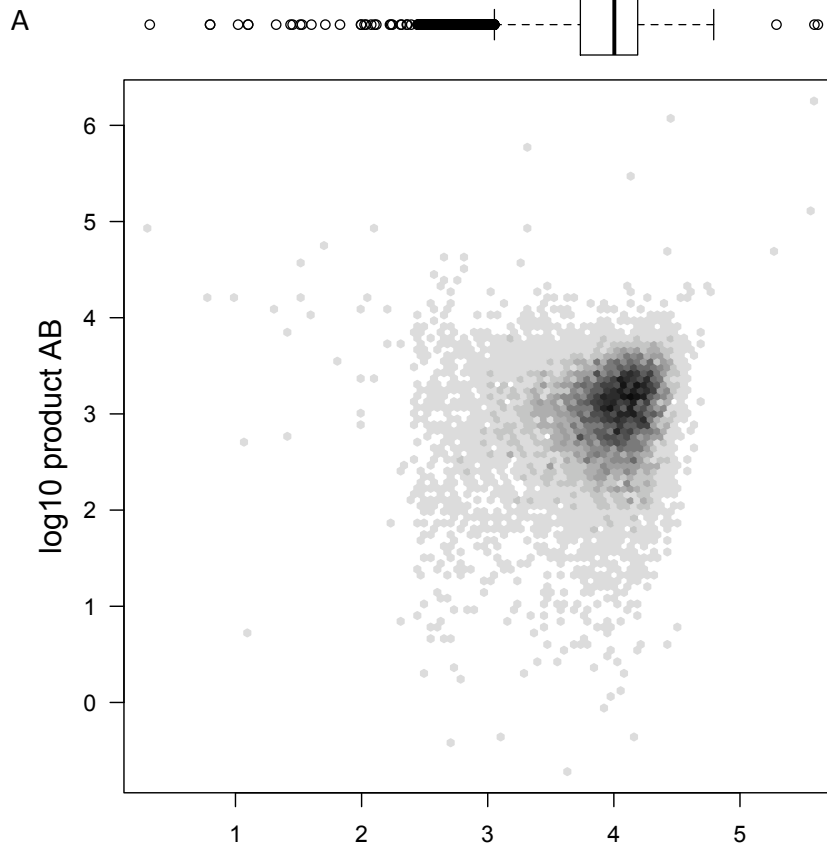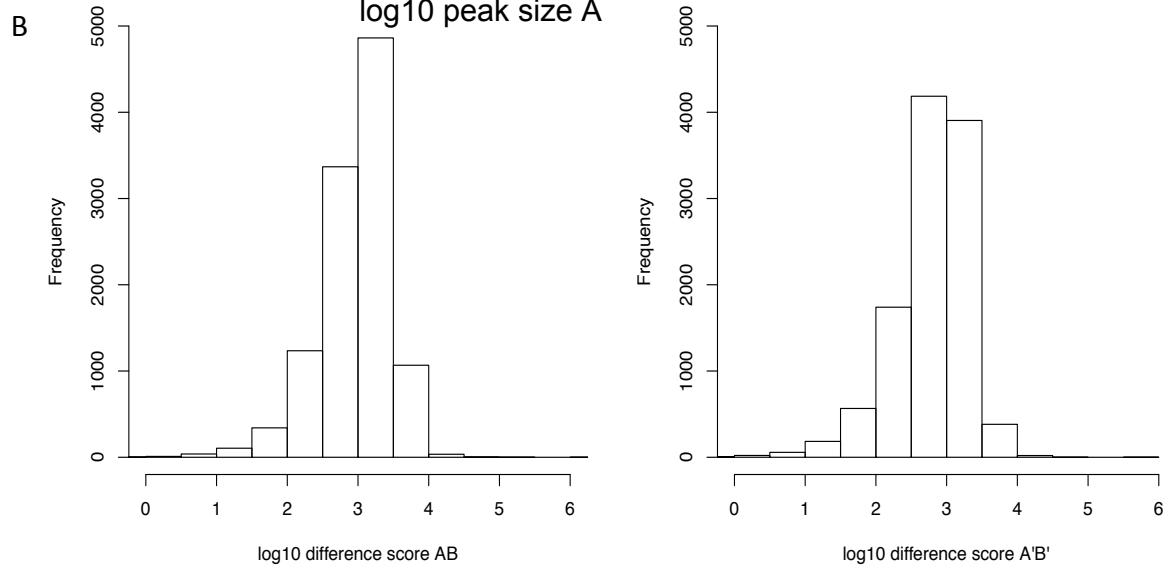

Supplement: Figure S7 — Difference scores. (A) Difference score as function of H3K4me3 peak sizes. Data values at 11,995 loci (comparisons between populations A and B) are clustered in hexbins which range from 1 to 64 (increase in greyness). Box-whisker plots show median, quantiles Q1, Q3, and outliers. (B) Histograms of difference scores of the comparison between control and treatment (AB) and between the offspring populations (A′B′). The 11,070 data points were selected by omitting minimal peaks as defined by the lower whisker of the peak size distribution boxplot from population A, see (A). (PDF) [file pone.0097568.s007.pdf]

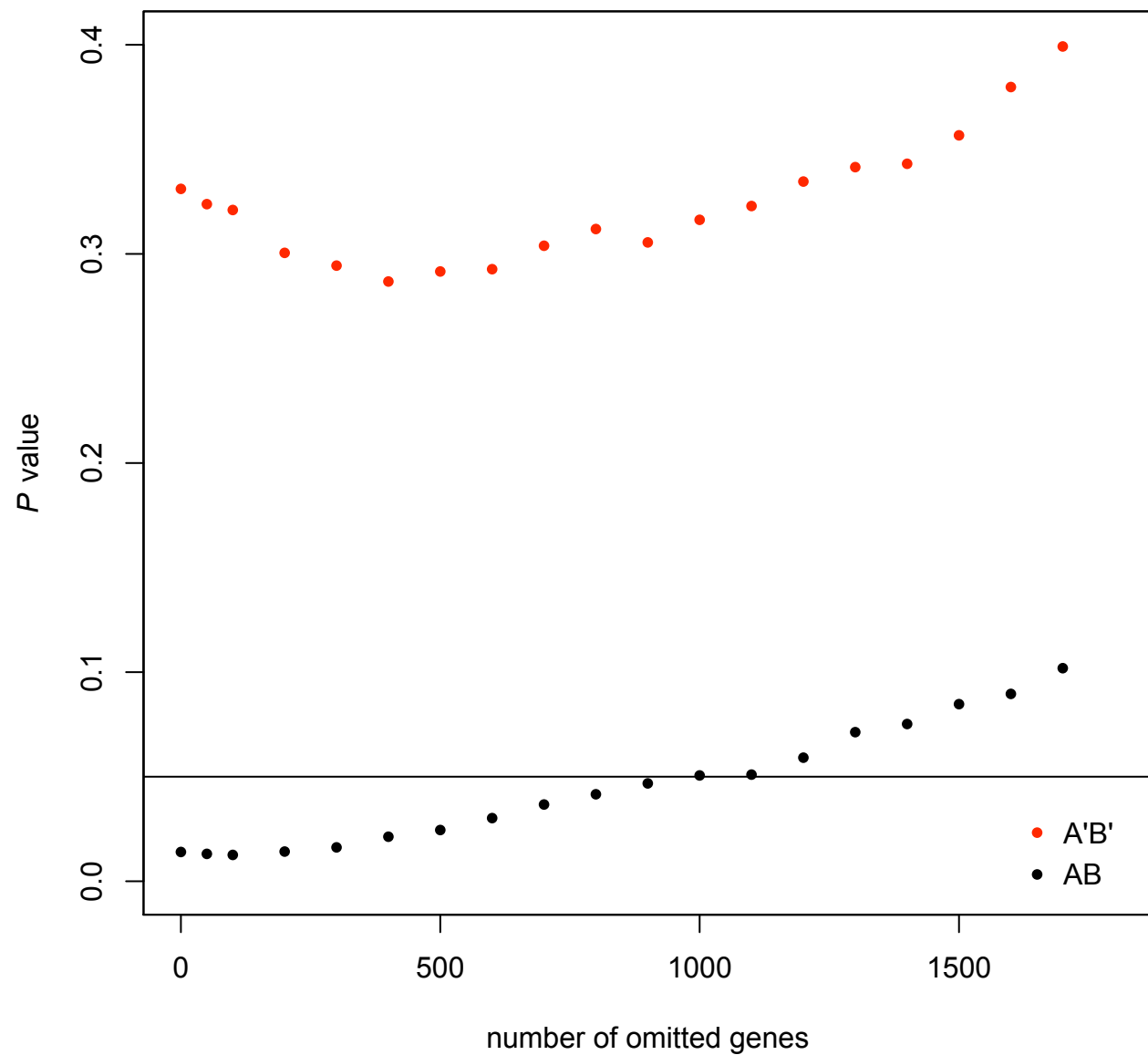

Supplement: Figure S8 — Mann-Whitney-Wilcoxon Test of reduced datasets after repeated omission of the most different loci. H3K4me3 marked genes were ranked by the absolute value of the difference score AB and genes with the largest scores were consecutively omitted from the gene pool. P values of the original and the reduced gene pool datasets were obtained by a Mann-Whitney-Wilcoxon Test. Black dots show the comparisons between populations A and B, red dots between A′ and B′. The horizontal line is drawn at the P value of 0.05. (PDF) [file pone.0097568.s008.pdf]

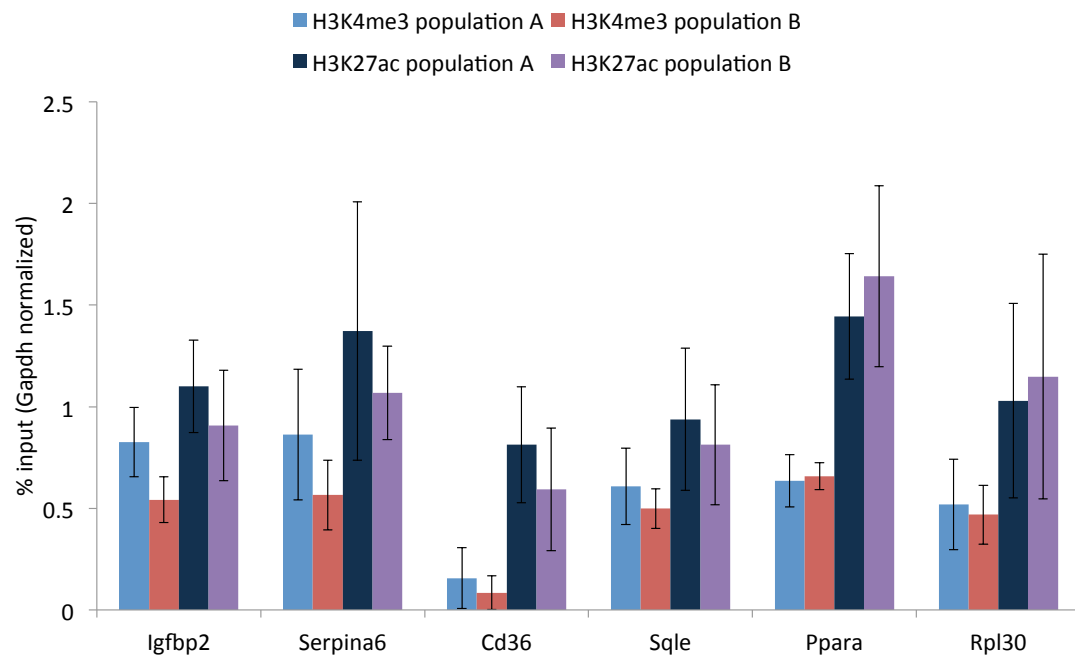

Supplement: Figure S9 — qPCR measurements of H3K4me3 and H3K27ac enriched ChIP-DNA. Individuals were eight young males from populations A and B, respectively, and shown are means and standard deviation. The Gapdh markings were lower in the H3K27ac ChIP-DNA compared to H3K4me3; therefore the normalized values appear to be larger. (PDF) [file pone.0097568.s009.pdf]

A

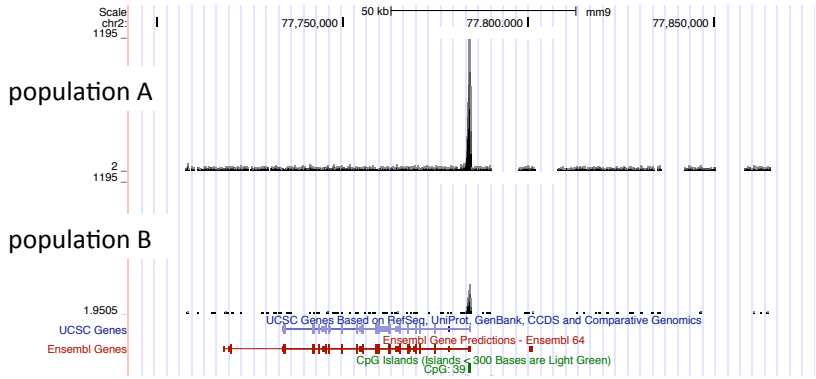

B

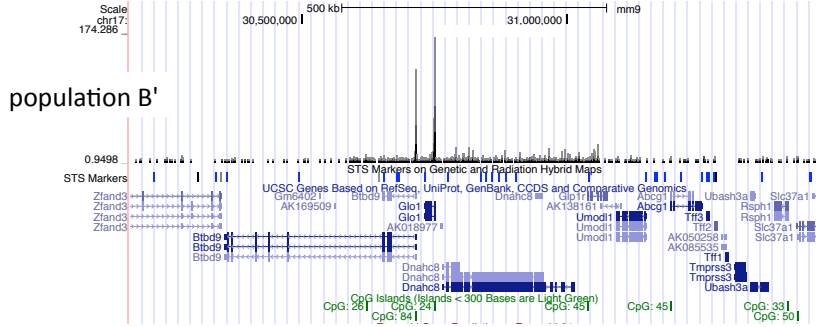

C

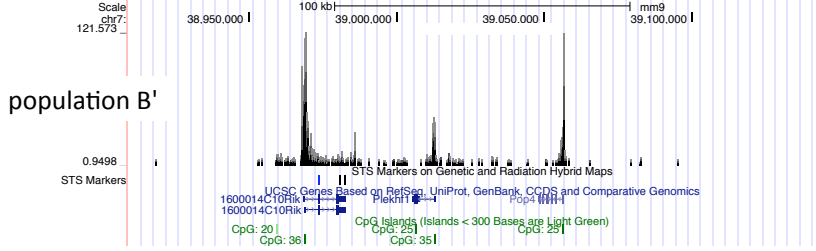

Supplement: Figure S10 — Copy number variant regions detected in our wild mouse population. H3K4me3 tracks with a local increase in background reads indicate copy number variant loci. (A) The background of the chromosomal region chr2∶77,707,909–77,866,330 around spliceosome factor Cwc22 was massively increased in population A. (B) The region chr17∶30,590,650–31,061,925 containing the genes Btbd9 and Glo1, both upmarked by H3K4me3 in population B, is a copy number variant. Shown are tracks from population B′ as the increase in background reads was most noticeable here. (C) The three genes 1600014CRik, Plekhf1, and Pop4 were upmarked in population B and the increase in background (chr7∶38,959,201–39,058,174), again best demonstrated in population B′, points to a copy number variant region. The difference scores AB of genes 1600014CRik and Pop4 were higher than the 99%tile of the difference score distribution. (PDF) [file pone.0097568.s010.pdf]
