# Supplementary material for: Genome-Wide Quantitative Analysis of Histone H3 Lysine 4 Trimethylation in Wild House Mouse Liver: Environmental Change Causes Epigenetic Plasticity
Source: PLoS One. 2014 May 21;9(5):e97568. doi: 10.1371/journal.pone.0097568 (PMC4029994; doi:10.1371/journal.pone.0097568)
Supplement: Figure S3 — Dependence of various difference measures on peak size. Three difference measures were calculated between population A and B at 11,995 genes. In this Figure, absolute difference was calculated as A–B for A>B and B–A for A<B. Fold change was calculated as A/B for A>B and B/A for A<B. The difference score is the product between difference and fold change. (PDF) [file pone.0097568.s003.pdf]

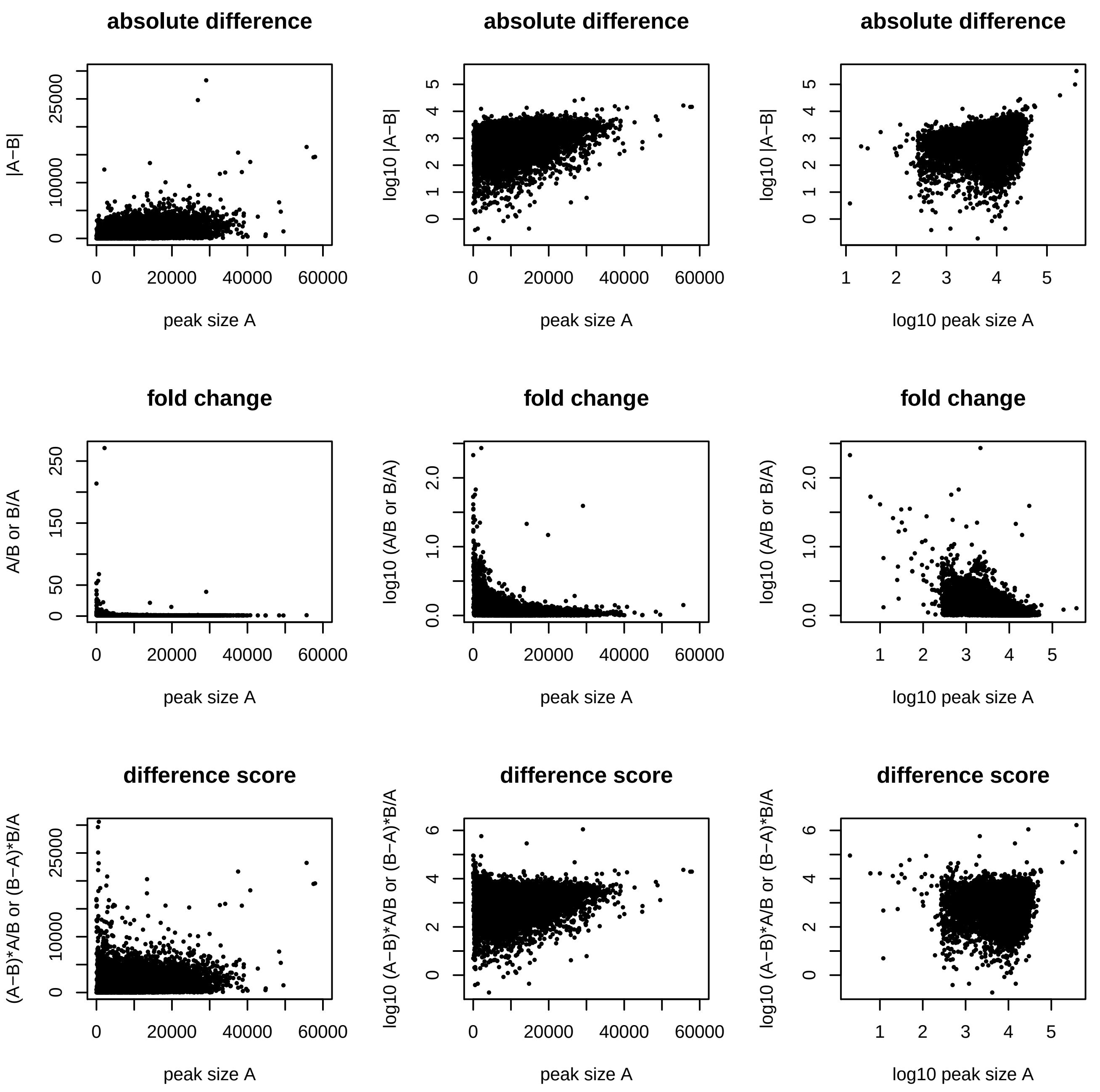
absolute difference
|A−B|
log10 |A−B|
peak size A
log10 peak size A
fold change
A/B or B/A
log10 (A/B or B/A)
difference score
(A−B)*A/B or (B−A)*B/A
log10 (A−B)*A/B or (B−A)*B/A
0
20000
40000
60000
10000
25000
1
2
3
4
5
50
150
250
0.0
1.0
2.0
6
