# Supplementary material for: Genome-Wide Quantitative Analysis of Histone H3 Lysine 4 Trimethylation in Wild House Mouse Liver: Environmental Change Causes Epigenetic Plasticity
Source: PLoS One. 2014 May 21;9(5):e97568. doi: 10.1371/journal.pone.0097568 (PMC4029994; doi:10.1371/journal.pone.0097568)
Supplement: Table S1 — Sequencing, mapping, and normalization factors for the experimental populations A and B and the respective offspring populations A′ and B′. (PDF) [file pone.0097568.s011.pdf]

Table S1. Sequencing, mapping, and normalization factors for the experimental populations A and B and the respective offspring populations A' and B'

	A	B	A'	B'
**sequence reads**	44,860,778	45,748,016	39,701,327	38,515,061
**uniquely mapped reads**	27,210,806 (60.66%)	27,901,537 (58,80%)	28,562,044 (71.94%)	28,479,574 (73.94%)
**unalignable reads**	9,580,791 (21.37%)	10,096,077 (22.07%)	3,882,210 (9.78%)	2,710,235 (7.04%)
**reads suppressed by uniqueness command**	8,069,181 (17.99%)	8,750,412 (19.13%)	7,257,073 (18.28%)	7,325,252 (19.02%)
**normalization factor "housekeeping genes"**	1	1.012	0.655	0.907
