# Supplementary material for: Genome-Wide Quantitative Analysis of Histone H3 Lysine 4 Trimethylation in Wild House Mouse Liver: Environmental Change Causes Epigenetic Plasticity
Source: PLoS One. 2014 May 21;9(5):e97568. doi: 10.1371/journal.pone.0097568 (PMC4029994; doi:10.1371/journal.pone.0097568)
Supplement: Table S2 — Peak size distribution of the normalized dataset. (PDF) [file pone.0097568.s012.pdf]

Table S2. Peak size distribution of the normalized dataset

	Min	1st Qu.	Median	Mean	3rd Qu.	Max
**33,940 peaks at annotated transcripts**						
**A**	2	5354	9678	10700	14730	386300
**B**	8	5203	9404	10450	14510	462000
**A'**	17	5798	9476	10410	13800	459900
**B'**	11	5553	9441	10340	13880	233600
**11,070 genes**						
**A**	1070	6141	10210	11240	14940	386300
**B**	1068	5957	9891	11050	14800	462000
**A'**	1091	6500	9852	10800	13980	459900
**B'**	1074	6298	9815	10780	14090	233600

33,940 peaks at annotated transcripts (ENSMUST, coverages > 0 in all four samples) were extracted from the database. Distribution of peak sizes at 11,070 genes with peak sizes larger than 1,068 in at least one of the datasets (see Methods).
