# Supplementary material for: Genome-Wide Quantitative Analysis of Histone H3 Lysine 4 Trimethylation in Wild House Mouse Liver: Environmental Change Causes Epigenetic Plasticity
Source: PLoS One. 2014 May 21;9(5):e97568. doi: 10.1371/journal.pone.0097568 (PMC4029994; doi:10.1371/journal.pone.0097568)
Supplement: Table S3 — Four moments of data distributions. (PDF) [file pone.0097568.s013.pdf]

Table S3. Four moments of data distributions

	A	B	A'	B'	log A	log B	log A'	log B'	score AB	score A'B'	log10 score AB	log10 score A'B'
**mean**	11243	11052	10803	10784	3.96	3.95	3.96	3.96	1664	1091	2.960508	2.791719
**sd**	8369	8164	7427	6710	0.298	0.298	0.255	0.270	15936	5112	0.5045024	0.5185522
**skewness**	16	17	22	7	-0.64	-0.58	-0.49	-0.55	102	95	-1.131844	-1.159240
**kurtosis**	659	880	1252	191	0.29	0.15	0.31	0.23	10658	9619	3.023096	2.889109

Distribution of TSS peak sizes and their logarithmic transformations of populations A, B, A', and B' and distribution of difference scores AB and A'B' (absolute values) and their logarithmic transformations.
