# Supplementary material for: Genome-Wide Quantitative Analysis of Histone H3 Lysine 4 Trimethylation in Wild House Mouse Liver: Environmental Change Causes Epigenetic Plasticity
Source: PLoS One. 2014 May 21;9(5):e97568. doi: 10.1371/journal.pone.0097568 (PMC4029994; doi:10.1371/journal.pone.0097568)
Supplement: Table S4 — Spearman’s rank correlation rho of H3K4me3 marking comparisons between the experimental populations (AB) and the offspring (A′B′). (PDF) [file pone.0097568.s014.pdf]

**Table S4.** Spearman's rank correlation rho of H3K4me3 marking comparisons between the experimental populations (AB) and the offspring (A'B')

| datasets | AB | A'B' |
|---|---|---|
| TSS (11,995 loci) | 0.9755 | 0.9839 |
| TSS (11,070 loci) | 0.9715 | 0.9806 |
| CpG (8,954 locations) | 0.9737 | 0.9842 |
| high expression (5,653 loci) | 0.9701 | 0.9793 |
| medium expression (4,441 loci) | 0.9700 | 0.9796 |
| low expression (2,772 loci) | 0.9707 | 0.9815 |
| differential expression (159 loci) | 0.9580 | 0.9719 |

Comparison at TSS and at CpG islands included all marked loci; in the TSS dataset of 11,070 loci, small peaks were omitted (see Methods). Comparison at high expressed, medium expressed, low expressed, or differentially expressed genes were at H3K4me3-marked TSS.
