# Supplementary material for: Genome-Wide Quantitative Analysis of Histone H3 Lysine 4 Trimethylation in Wild House Mouse Liver: Environmental Change Causes Epigenetic Plasticity
Source: PLoS One. 2014 May 21;9(5):e97568. doi: 10.1371/journal.pone.0097568 (PMC4029994; doi:10.1371/journal.pone.0097568)
Supplement: Table S5 — P values of peak size distributions from comparisons of population A with B and A′ with B′. (PDF) [file pone.0097568.s015.pdf]

Table S5. *P* values of peak size distributions from comparisons of population A with B and A' with B'

	TSS peaks		marked CpG islands	
**test method**	**A - B**	**A' - B'**	**A - B**	**A' - B'**
wilcox.test (median)	0.0140	0.3311	0.4359	0.6656
Monte Carlo (median)	0.0047	0.7725	0.5829	0.7962
Monte Carlo (variance)	0.9235	0.8741	0.6933	0.7218

wilcox.test was performed in R; Monte Carlo simulations were performed with 100,000 iterations.
