# Supplementary material for: Genome-Wide Quantitative Analysis of Histone H3 Lysine 4 Trimethylation in Wild House Mouse Liver: Environmental Change Causes Epigenetic Plasticity
Source: PLoS One. 2014 May 21;9(5):e97568. doi: 10.1371/journal.pone.0097568 (PMC4029994; doi:10.1371/journal.pone.0097568)
Supplement: Table S6 — Primer sequences for qPCR measurements. (PDF) [file pone.0097568.s016.pdf]

Table S6. Primer sequences for qPCR measurements at TSS

**gene**	**forward primer 5'-3'**	**reverse primer 5'-3'**
Cd36	ACTTGTGGCAAACAGGGCTGGAG	AGTCCGATGAGAGAGTGCAAGGCC
Cdkn1a	TGAGCCCAGTGGGTGTCCCCAGA	GGACGCTCAGACCCACACCCGT
Gapdh	GCACCAGCATCCCTAGACC	GTGCAGTGCCAGGTGAAAAT
Igfbp2	CTGTGAGCACTAGTTTTGGGCTTG	CTCTCTTTTCCCACGTGAGAGTCA
Insig2	CTGCGGAGGGCAGGCTGAAGAA	CCTCCCCTCTTCCCTCCACTCATCC
Plin5	GCAGGTCGCCTACCAAAGGCTGC	TGCAGGTCACCCAAACAGCCCCC
Ppara	GTCTGGAGACCCACAGCCACT	AAACAGCTGCGAACACCAATGT
Rpl30	TACCTTTGAGGCACGTTGTGTGAC	ACCATCACCTCCATAATCCAGCCA
Rpp21	TCTGTGTGGCAGTAAAACCTAGCC	AGCTTCCTGTACCAGGTGAGACG
Serpina6	AAAAATCTCAAGCAAGCAGGCACT	AGACGTGAGTCACCCCTGACAGTA
Slc38a3	GCAAGATTCTGACTCTCGGAAAGC	ACAATACCTCCCCCAGTGCATAGT
Sqle	TGGTGACAAAAGACCGTTTACAGC	CAACGGCTCCTGATTACACACTTC
Tgfbr2	CCCCAGTGAACCCCCAGGGTCGC	CCTGTGGACGCGCATCGCCAGC
