# Supplementary material for: Genome-Wide Quantitative Analysis of Histone H3 Lysine 4 Trimethylation in Wild House Mouse Liver: Environmental Change Causes Epigenetic Plasticity
Source: PLoS One. 2014 May 21;9(5):e97568. doi: 10.1371/journal.pone.0097568 (PMC4029994; doi:10.1371/journal.pone.0097568)
Supplement: Text S1 — Supporting Methods and Results. (PDF) [file pone.0097568.s017.pdf]

Text S1. Supporting Methods and Results

METHODS

Chromatin preparation and immunoprecipitations: To cross-link DNA with histone proteins, 100 to 120 mg of frozen mouse liver were placed into a 2 ml tube containing PBS/formaldehyde (1%) and cut for 1 min with fine scissors into small pieces (1 mm^3). Tubes were left at room temperature for another 9 min, then briefly centrifuged and the supernatant was removed. Cross-linking was stopped by adding 125 mM glycine (in PBS) and samples were incubated for 10 min on a rotating wheel. After centrifugation, the tissue was washed twice in cold PBS containing 1 mM PMSF before adding lysis buffer (buffer A, SimpleChIP™ Enzymatic Chromatin IP Kit, Cell Signaling, Beverly, MA). The samples were homogenized on ice in a 5 ml Dounce homogenizer (10 strokes with pestle A and 10 strokes with pestle B) and left on ice for another 10 min. The procedure then followed the protocol of the ChIP Kit: Cell lysates were treated with micrococcal nuclease for 20 min at 37° and aliquots of 500 µl were sonicated in 1.5 ml Eppendorf tubes for 3 - 4 x 20 sec on ice with 1 min resting intervals at an amplitude of 70 - 80%. After centrifugation at 10,000 rpm for 10 min at 4° in a microcentrifuge, soluble chromatin was shock frozen on dry ice and transferred to -80° for storage. The chromatin DNA concentration was determined with the Quant-iT™ dsDNA Broad-Range Assay Kit (Life Technologies, Carlsbad, CA) using a NanoDrop 3300 Fluorospectrometer.

For sequencing, 8 chromatin samples were pooled using 2 µg DNA of chromatin per animal and, after pre-clearing with a small amount of beads, immunoprecipitations were performed on a rotating platform at 4° over night with 10 µl of trimethyl-histone H3 (Lys4) rabbit monoclonal antibody (#9751, Cell Signaling). For quantitative PCR measurements, 2 µg of chromatin per sample was immunoprecipitated with 2 µl of the anti-H3K4me3 antibody or 0.5 µl of anti-rabbit IgG control antibody (#2729, Cell Signaling). Immunoprecipitates were captured on agarose beads using a rotating wheel at 4° for at least 3 h. Input samples (2%) were taken from the precleared lysates and stored at -20° until needed. Immunoprecipitates were washed 3 times in ice-cold 1 ml of low salt ChIP-wash buffer and 1 times in 1 ml of high salt ChIP-wash buffer. After careful aspiration of the wash solution, beads were resuspended in 150 µl of elution buffer and incubated at 65° with gentle mixing (1,200 rpm) for at least 30 min. The supernatant was treated with 2 µl of proteinase K (20 mg/ml) at 65° for 2 h to digest histones and antibody, followed by spin column purification of DNA (MinElute PCR Purification Kit, Quiagen, Hilden, Germany). DNA was eluted in 2 x 35 µl TElow (10 mM Tris, pH 8, 0.1 mM EDTA) and stored at -20°.

Sequencing and Mapping: DNA libraries for single-read sequences (36 bp length) were prepared using 10 ng of ChIP DNA and the ChIP-Seq DNA Sample Prep Kit (#IP-102-1001, Illumina, San Diego, CA) following the manufacturer's instructions. Sequences were obtained on an Illumina Genome Analyzer IIx and PhiX was used as control for each run. Samples from populations A and B were run on two lanes each and resulted in approx. 45,000,000 sequence reads (Table S1). Samples from populations A' and B' were applied to only one lane and gave nearly 40,000,000 reads each. 36 bp reads were mapped onto the Mouse Reference Genome mm9 using bowtie [1] with unique mapping, allowing two mismatches, and applying the "best" switch. To be able to compare our wild mouse data

with C57BL/6 liver data, we repeated the mapping for the H3K4me3 mouse liver dataset published by Robertson et al. [2]. Due to the large coverage in our experiment (6 to 7 times higher than described in Robertson et al. 2008), we did not employ the iterative alignment approach described by Robertson et al. [3]. The coverage per nucleotide was initially normalized by the number of mapped reads per sample (Table S1).

Normalization: After inspecting browser tracks of the four populations, it became clear that the normalization by the number of mapped reads was not sufficient for a quantitative analysis. The samples of populations A' and B' were prepared and sequenced 12 months after those of populations A and B, and we found more small read clusters outside annotated TSS dispersed throughout the genome (Figure S2A). For a quantitative analysis of peak sizes, the total number of reads contributing to functional peaks therefore needed to be adjusted. Vandesompele et al. [4] tested several housekeeping genes for transcription studies and proposed to normalize expression with respect to a set of genes with independent cellular functions. We selected nine housekeeping genes that were maximally similar in all our samples with peak sizes ranging from 9,000 to 38,000 (population A). After normalization, peak size correlations were improved (Figure S2B). The normalization factors were very similar to an alternative normalization procedure where the factor was calculated from the sum of all reads that contribute to TSS peaks. For the normalization of CpG islands spanning H3K4me3 peaks, the respective sum of all reads mapping to CpG islands was used. The normalization factors were 1.0887 (B), 0.6961 (A'), and 0.9011 (B').

Manual curation of exact peak sizes for candidate loci: Genes with the largest difference scores (AB comparison) were selected by the 99%tile threshold (absolute value of difference score: 6725.5) which resulted in a list of 111 loci. After the elimination of regions where there were no clear TSS peaks (background of higher copy number genes; 3 loci) or where peak shapes were unlike the typical TSS marking (clusters of extremely large peaks within repetitive regions; 11 loci), the validity of differently marked genes was assessed by manually checking peak locations as displayed in our custom UCSC genome browser tracks. Exact difference scores were re-calculated for the entire peak window (exact peaks) and genes with scores under the value of the 99%tile were eliminated. These were genes with either bidirectional promoters where the H3K4me3 marking overlapped TSS annotations of two different genes (for example *Gnl3/Pbrm1*, *Psma7/Ss18l1*, *Abi3/Gngt2*) or poorly marked genes if the exact peak window did not agree with the TSS±1000 bp computation (*Kdr, Trim44*). The final list contained 77 genes. These manual corrections were not performed for the much larger gene lists submitted to KEGG [5] or GO [6] enrichment analyses because with decreasing difference scores, the possible mismatch between exact peak sizes and the TSS±1000 bp window computation dropped rapidly.

RESULTS

H3K4me3 enrichment: To assess the quality of our H3K4me3 data obtained from wild mice, we compared our H3K4me3 profile with similar data from inbred C57BL/6 mice [2]. Of 82,813 annotated transcripts (ENSEMBL mouse database m37), 35,156 (population A), 35,198 (population B), 47,518 (population A'), and 50,598 (population B') were marked by H3K4me3. This corresponded to peaks at

12,416 (A), 12,473 (B), 16,517 (A'), and 17,541 (B') RefSeq genes. The H3K4me3 liver dataset from the reference mouse C57BL/6 [2] contains 32,100 marked TSS (corresponding to 11,391 genes).

Markings at typical liver genes were qualitatively very similar between our samples and the reference dataset. Of 3,990 liver genes from the UniProtKB Database that matched RefSeq genes, 74% were marked in population A (75% in population B) and 70% in the C57BL/6 dataset. 68% of UniProtKB metabolic genes (696) were marked in population A and B; 65% in C57BL/6. Of 677 genes annotated with the phenotype "Liver and Bile" (table jaxPhenotypes of database mm9) that matched RefSeq genes, 69% were marked in population A (70% in population B). In contrast, only 36% of genes associated with "Taste/Smell" and 40% of genes associated with "Touch" were marked in our liver samples (26% and 32%, respectively, for the published inbred mouse dataset). These gene sets include cell-type independent factors such as intracellular signaling components and genes involved in growth, transcription, translation, and protein modification. The number of peaks found outside of annotated transcripts was very small (18 locations in population A and 20 in population B) with most of the aggregate coverages smaller than 1,000. We conclude from functional gene annotation analyses and comparisons with the published inbred mouse liver dataset that our wild-mouse ChIP-Seq dataset is of high quality and suitable for further analysis.

Qualitative differences between the two experimental populations A and B were negligible as judged by the number and size of peaks present in one population and absent in the other (exclusive peaks). 635 genes were marked exclusively in population A and not in B, but peak sizes were rather small (maximal peak size: 2,619; average peak size: 613). Similarly, 666 genes were marked in population B but not in A (maximum: 3,050; average: 607).

Genome-wide peak size distribution: H3K4me3 peak sizes at all marked transcripts varied between a few hundred reads to over 200,000 (Table S2). They were not normally distributed, even after logarithmic transformation. All datasets showed a skewness to the right (or to the left after log transformation) and tailing (Table S3).

CpG islands: With the exception of one poorly covered CpG island, the 1% most differently marked CpG island locations coincided with annotated gene promoter regions, thus complementing our TSS search, but not adding extra information. Differences between A and B were often caused by a shift in the position of dismantled nucleosomes as visible from a wider gap at the TSS in one sample or a small shift of peaks just outside the CpG window (not shown). In conclusion, differences in the peak size distributions of the two experimental populations were readily detected at TSS markings.

Literature Cited

1. Langmead B, Trapnell C, Pop M, Salzberg SL (2009) Ultrafast and memory-efficient alignment of short DNA sequences to the human genome. Genome Biol 10: R25.
2. Robertson AG, Bilenky M, Tam A, Zhao Y, Zeng T, et al. (2008) Genome-wide relationship between histone H3 lysine 4 mono- and tri-methylation and transcription factor binding. Genome Res 18: 1906-1917.
3. Robertson G, Hirst M, Bainbridge M, Bilenky M, Zhao Y, et al. (2007) Genome-wide profiles of STAT1 DNA association using chromatin immunoprecipitation and massively parallel sequencing. Nat Methods 4: 651-657.

4. Vandesompele J, De Preter K, Pattyn F, Poppe B, Van Roy N, et al. (2002) Accurate normalization of real-time quantitative RT-PCR data by geometric averaging of multiple internal control genes. Genome Biol 3: RESEARCH0034.
5. Kanehisa M, Araki M, Goto S, Hattori M, Hirakawa M, et al. (2008) KEGG for linking genomes to life and the environment. Nucleic Acids Res 36: D480-484.
6. Ashburner M, Ball CA, Blake JA, Botstein D, Butler H, et al. (2000) Gene ontology: tool for the unification of biology. The Gene Ontology Consortium. Nat Genet 25: 25-29.
