# Supplementary material for: Genome-Wide Quantitative Analysis of Histone H3 Lysine 4 Trimethylation in Wild House Mouse Liver: Environmental Change Causes Epigenetic Plasticity
Source: PLoS One. 2014 May 21;9(5):e97568. doi: 10.1371/journal.pone.0097568 (PMC4029994; doi:10.1371/journal.pone.0097568)
Supplement: File S1 — Genes with the largest H3K4me3 differences between populations A and B. (PDF) [file pone.0097568.s018.pdf]

Genes with the largest H3K4me3 differences between populations A and B

sym	score_AB	score_A'B'	exact_score_AB	exact_score_A'B'	TSS_info; CNVs	full_gene_name	function	functional_category	expression_level	CpG
Btbd9	21675	12636	23417	change retained in offspring by 60%	CNV	BTB (POZ) domain containing 9	protein-protein interaction	Cell adhesion	high	84
Slc11a2	8459	2077	10133	change lost	ENSMUST00000140535	solute carrier family 11 (proton-c	ion transmembrane transporter	Cell cycle: apoptosis	medium	---
Dusp1	15561	-3781	18775	change lost		dual specificity phosphatase 1	phosphatase activity	Cell cycle: apoptosis, positive regulation	high	103
Ddit4	7949	-3061	11914	change lost		DNA-damage-inducible transcrip	14-3-3 protein binding	Cell cycle: apoptotic process; response to	high	92
Gadd45g	9100	1381	14873	change lost		growth arrest and DNA-damage-	activation of the p38/JNK pathwa	Cell cycle: apoptotic process; response to	high	139
Cdkn1a	85496	3634	79188	change lost		cyclin-dependent kinase inhibito	Regulator cell cycle at G1	Cell cycle: arrest	medium	157
Rad54l2	7260	3359	7267	change retained in offspring by 44%		RAD54 like 2 (S. cerevisiae)	transcription cofactor activity; he	Cell cycle: proliferation, chromatin remoc	medium	44
Mgst1	6869	329	7705	change lost		microsomal glutathione S-transfe	reduction of lipid hydroperoxides	ER: protection of membrane from oxidati	high	---
Herpud1	7330	10340	21361	change retained in offspring by 52%	bidirectional promotor (Gm15890)	homocysteine-inducible, endopla	ubiquitin-dependent protein cata	ER: response to unfolded protein	high	62
Lbp	7407	729	11007	change lost		lipopolysaccharide binding prote	protein binds LPS and interacts w	Immune: acute defense response	high	---
Uba5	15663	3523	10588	change retained in offspring by 29%	TSS window overlapping with Acad11	ubiquitin-like modifier activating	ubiquitination and proteasome d	Immune: antigen processing	high	45
Cd97	8213	-508	9516	change lost		CD97 antigen	EGF-TM7 subfamily of adhesion (	Immune: cell adhesion; inflammatory res	high	33
Serpina3k	8343	-850	11470	change lost	overlaps with Serpina3m	serine (or cysteine) peptidase inh	plasma protease inhibitor	Immune: inflammation	high	---
Serpina3m	8343	-850	12292	change lost	overlaps with Serpina3k	serine (or cysteine) peptidase inh	plasma protease inhibitor: Alpha	Immune: inflammation	high	---
Agxt	9755	1822	13562	change lost		alanine-glyoxylate aminotransfer	glycine biosynthesis peroxisome	Metabolism: amino acid metabolism; alai	high	---
Gnmt	8855	-2316	18801	change lost		glycine N-methyltransferase	catalyzes the conversion of S-ade	Metabolism: amino acid metabolism; gly	high	20
Hpd	8427	2003	11709	change lost		4-hydroxyphenylpyruvic acid dio:	oxidoreductase activity	Metabolism: amio acid metabolism, tyros	high	---
Cyp4a14	20790	631	40383	change lost		cytochrome P450, family 4, subfa	arachidonic acid monooxygenase	Metabolism: arachidonic acid metabolic	high	---
Cyp2d22	8249	1238	35327	change lost		cytochrome P450, family 2, subfa	arachidonic acid monooxygenase	Metabolism: arachidonic acid metabolic	high	---
Cyp4f14	7088	-2743	8164	change lost		cytochrome P450, family 4, subfa	arachidonic acid monooxygenase	Metabolism: arachidonic acid metabolic	high	---
Glo1	47651	12674	62040	change retained in offspring by 27%	CNV	glyoxalase 1	methylglyoxal degradation	Metabolism: carbohydrate metabolism;	high	24
Ppp1r3b	10086	3621	22243	change lost		protein phosphatase 1, regulator	Insulin signalling pathway	Metabolism: carbohydrate; glycogen met	medium	29
Ppp1r3c	7241	-302	10993	change lost		protein phosphatase 1, regulator	a regulatory subunit of protein pl	Metabolism: carbohydrate; glycogen met	high	37
Npas2	11082	4979	7545	change retained in offspring by 71%		neuronal PAS domain protein 2	transcription factor	Metabolism: cellular lipid metabolic proc	NA	103
Insig2	12967	5281	29234	change retained in offspring by 32%		insulin induced gene 2	essential for the feedback inhibit	Metabolism: cholesterol biosynthetic pro	high	---
Fmo5	7076	1817	20756	change lost		flavin containing monooxygenase	monooxygenase activity	Metabolism: drug metabolism - cytochro	high	---
Acad11	15897	3917	9229	change lost	TSS window overlapping with Uba5	acyl-Coenzyme A dehydrogenase	oxidoreductase activity	Metabolism: fatty acid beta-oxidation	low	45
Es31	8841	757	8841	change lost		carboxylesterase 3A (Ces3a)	hydrolysis or transesterification c	Metabolism: fatty acyl and cholesterol es	NA	---
Mthfr	7809	-937	10872	change lost	bidirectional promotor (Clcn6)	5,10-methylenetetrahydrofolate	conversion of 5,10-methylenetet	Metabolism: folic acid metabolic process	medium	73
G6pc	8259	459	17344	change lost		glucose-6-phosphatase, catalytic	hydrolysis of D-glucose 6-phosph	Metabolism: glucose homeostasis	high	---
Slco1a1	12403	-1523	19640	change lost		solute carrier organic anion trans	bile acid/salt transporter	Metabolism: lipid and lipoprotein	high	---
Etnk2	15574	-1006	22322	change lost		ethanolamine kinase 2	kinase activity	Metabolism: lipid metabolism; glyceroph	medium	71
Plin5	15580	211	23096	change lost		perilipin 5 = Lsdp5	LSDP5 is stimulated by ligand acti	Metabolism: lipid storage	high	---
Dbi	8908	-154	17086	change lost	bidirectional promotor (3110009E18Rik)	diazepam binding inhibitor	acyl-CoA-binding protein: acyl-Co	Metabolism: lipid. PPAR signaling	high	35
Nr0b2	9801	2911	14919	change lost		nuclear receptor subfamily 0, grc	tyrosine phosphatase Shp-1	Metabolism: regulation of insulin secretic	medium	---
Cyp2e1	7091	2748	7242	change retained in offspring by 44%		cytochrome P450, family 2, subfa	monooxygenase activity	Metabolism: triglyceride metabolic proce	high	---
Slc25a25	7542	-2343	8746	change lost	ENSMUST00000153886	solute carrier family 25 (mitocho	calcium transmembrane transpor	Mitochondrion: calcium transporter	high	---
1810011O10Rik	7591	-2136	8789	change lost		thyroid cancer-1	May decrease apoptosis (By simil	ND	high	---
1600014C10Rik	18311	67904	27459	change retained in offspring > 100%	CNV (genomic vicinity to Plekhf1 and Pop4)	hypothetical protein LOC72244	ND	ND	high	36
Pnkd	16550	-1640	22013	change lost		paroxysmal nonkinesiogenic dysk	hydroxyacylglutathione hydrolase	ND	high	38
Dscr3	7460	-450	8258	change lost		Down syndrome critical region ge	vacuolar transport	ND	high	99
AC124005.1	7047	-642	6996	change lost	chr3:123,210,238-123,212,243	region of SNORA24	ND	ND	NA	76
Anubl1	6859	5068	9484	change retained in offspring by 94%	Zfand4	AN1, ubiquitin-like, homolog (Xe	Zinc finger protein	ND	medium	---
Tmie	7425	-1137	11019	change lost	bidirectional promotor (Als2cl)	transmembrane inner ear	membrane protein	Sensory perception: sound	medium	57
AL592283.1	7233	-35	7233	change lost	bidirectional promotor (Nfib)	ENSMUST00000139835	transcription factor	Transcription: positive regulation	NA	---
Zfp36l1	7941	-601	8619	change lost		zinc finger protein 36, C3H type-l	zinc ion binding protein	Transcription/translation: mRNA stability	high	23, 42
Srrm4	11793	2035	11793	change lost	ENSMUST00000139425	serine/arginine repetitive matrix	RNA binding	Transcription/translation: RNA splicing	high	23
Pop4	12490	10837	12490	change retained in offspring by 89%	CNV (genomic vicinity to Plekhf1 and 1600014C10Rik)	processing of precursor 4, ribonu	one subunit of the small nucleola	Translation: RNA transport; ribosome bio	high	25
Spp2	-6732	-2869	-10209	change lost		secreted phosphoprotein 2	ND	Bone remodeling	high	---
Fnip2	-7303	1091	-11451	change lost	bidirectional promotor (ENSMUST00000091023)	folliculin interacting protein 2	regulation of protein phosphrylat	Cell cycle: apoptosis, DNA damage respoi	high	---
Ypel3	-8869	798	-8869	change lost		yippee-like 3 (Drosophila)	protein with grp	Cell cycle: apoptosis	high	56
Tnfaip2	-7082	-2524	-8797	change lost		tumor necrosis factor, alpha-indu	angiogenesis	Cell cycle: cell differentiation	high	44
Hjurp	-19558	-19389	-16068	change retained in offspring by 106%	CNV	Holliday junction recognition pro	CENP-A chromatin assembly facto	Cell cycle: centromeric core chromatin as	high	49
Igfbp2	-13736	4531	-13736	change lost		insulin-like growth factor binding	growth factor binding protein	Cell cycle: regulation of cell growth (inhib	high	32,20
Serpina6	-7667	-2807	-10349	change lost		serine (or cysteine) peptidase inh	alpha-globulin protein with cortic	Hormonal regulation: glucocorticoid tran	high	---
Dio1	-17776	-5716	-38703	change lost		deiodinase, iodothyronine, type I	oxidoreductase activity	Hormone biosynthetic process	high	---
Bcl3	-11260	-5732	-11260	change retained in offspring by 89%	ENSMUST00000139680	B-cell leukemia/lymphoma 3	transcriptional co-activator	Immune: antimicrobial humoral response	high	43
H2-L	-10206	-4056	-10206	change retained in offspring by 27%		histocompatibility 2, D region	MHC class I receptor activity	Immune: defense response	NA	115
Bcl6	-7903	-403	-9853	change lost	ENSMUST00000135352	B-cell leukemia/lymphoma 6	zinc finger transcription factor	Immune: negative regulation of type 2 im	high	---
Abcf1	-7227	1384	-7269	change lost		ATP-binding cassette, sub-family	ATP-binding cassette (ABC) trans	Inflammation: may be regulated by tumo	high	62
Ppp1r3g	-7192	-810	-7454	change lost		protein phosphatase 1, regulatory	(inhibitor) subunit 3G	Metabolism: carbohydrate; glycogen met	high	102
Acacb	-7556	1831	-8788	change lost	ENSMUST00000102582 (upstream gene TSS)	acetyl-Coenzyme A carboxylase b	carboxylation of acetyl-CoA to m	Metabolism: fatty acid biosynthesis (rate	low	---
Rnf186	-7134	-1253	-7934	change lost		ring finger protein 186	Zn finger protein	ND	medium	---
Pcp4l1	-10262	-307	-11819	change lost		Purkinje cell protein 4-like 1	ND	ND	high	25
Klhdc7a	-12305	-6294	-14652	change retained in offspring by 92%		kelch domain containing 7A	membrane protein	ND	low	21
E030030I06Rik	-13383	-1189	-12537	change lost		RIKEN cDNA E030030I06 gene; A	ND	ND	low	221
1110034G24Rik	-15218	5745	-15218	change lost	ENSMUST00000141460 (AL935270.6)	hypothetical protein LOC73747	ND	ND	high	---
AC132460.2	-20303	-4013	-20303	change lost	chr17:28,144,377-28,146,377	ENSMUST00000156965 (Anks1, 1	ND	ND	NA	---
Tgfbr2	-7836	137	-8388	change lost		transforming growth factor, beta	serine/threonine kinase receptor	Signal transduction: ser/thr kinase recept	high	83
Ralgps2	-8671	-283	-9200	change lost		Ral GEF with PH domain and SH3	guanyl-nucleotide exchange facto	Signal transduction: small GTPase mediat	high	112
Fzd8	-8502	1636	25536	change lost		frizzled homolog 8 (Drosophila)	Wnt-activated receptor activity	Signal transduction: Wnt singaling pathw	medium	218
Arid1a	-7328	-1483	-8179	change lost	ENSMUST00000008024	AT rich interactive domain 1A (S\	helicase and ATPase activities	Transcription regulation: chromatin orga	medium	30
Dbp	-10264	-632	-16956	change lost		D site albumin promoter binding	transcription factor	Transcription: positive regulation. circadi	high	26,19,49
SSU_rRNA_5	-10501	12740	-42155	change lost	near mir-715, highly marked region	ribosomal RNA, small subunit	ribosome	Transcription/translation: ribosome	NA	360, 81
Cwc22	-1662660	-520398	-1570329	change retained in offspring by 32%	CNV	CWC22 spliceosome-associated protein homolog (S. cerevisiae)		Transcription/translation: RNA/DNA bind	high	39
Elac1	-8361	142	-8361	change lost		elaC homolog 1 (E. coli)	endoribonuclease activity	Transcription/translation: tRNA processir	low	65
Rpp21	-15232	-1553	-15250	change lost		ribonuclease P 21 subunit (huma	ribonuclease P activity	Transcription/translation: tRNA processir	high	26
