# Supplementary material for: Genome-Wide Quantitative Analysis of Histone H3 Lysine 4 Trimethylation in Wild House Mouse Liver: Environmental Change Causes Epigenetic Plasticity
Source: PLoS One. 2014 May 21;9(5):e97568. doi: 10.1371/journal.pone.0097568 (PMC4029994; doi:10.1371/journal.pone.0097568)
Supplement: File S2 — KEGG and GO enrichment analysis. (PDF) [file pone.0097568.s019.pdf]

KEGG and GO enrichment analysis

77 most differently marked loci (71 user IDs mapped) against 11,070 marked genes

Significance Level: 0.05, Statistics Test: Hypergeometric, MTC: BH, Minimum: 3

**C**	number of reference genes in the category
**O**	number of genes in the gene set and also in the category
**E**	expected number in the category
**R**	Ratio of enrichment
**rawP**	p value from hypergeometric test (rawP)
**adjP**	p value adjusted by the multiple test adjustment (adjP)

Enriched KEGG pathways

**KEGG pathway----Drug metabolism - cytochrome P450----00982**			
C=38;O=4;E=0.28;R=14.28;rawP=0.0002;adjP=0.0014			
Cyp2d22	56448	ENSMUSG00000061740	cytochrome P450, family 2, subfamily d, polypeptide 22
Fmo5	14263	ENSMUSG00000028088	flavin containing monooxygenase 5
Mgst1	56615	ENSMUSG00000008540	microsomal glutathione S-transferase 1
Cyp2e1	13106	ENSMUSG00000025479	cytochrome P450, family 2, subfamily e, polypeptide 1
**KEGG pathway----Arachidonic acid metabolism----00590**			
C=25;O=3;E=0.18;R=16.27;rawP=0.0008;adjP=0.0028			
Cyp4a14	13119	ENSMUSG00000028715	cytochrome P450, family 4, subfamily a, polypeptide 14
Cyp4f14	64385	ENSMUSG00000024292	cytochrome P450, family 4, subfamily f, polypeptide 14
Cyp2e1	13106	ENSMUSG00000025479	cytochrome P450, family 2, subfamily e, polypeptide 1
**KEGG pathway----Insulin signaling pathway----04910**			
C=107;O=4;E=0.79;R=5.07;rawP=0.0079;adjP=0.0184			
Acacb	100705	ENSMUSG00000042010	acetyl-Coenzyme A carboxylase beta
Ppp1r3b	244416	ENSMUSG00000046794	protein phosphatase 1, regulatory (inhibitor) subunit 3B
G6pc	14377	ENSMUSG00000078650	glucose-6-phosphatase, catalytic
Ppp1r3c	53412	ENSMUSG00000067279	protein phosphatase 1, regulatory (inhibitor) subunit 3C

Enriched GO categories

**biological process----oxidation-reduction process----GO:0055114**			
C=597;O=17;E=4.44;R=3.83;rawP=1.15e-06;adjP=0.0005			
Cyp2d22	56448	ENSMUSG00000061740	cytochrome P450, family 2, subfamily d, polypeptide 22
Uba5	66663	ENSMUSG00000032557	ubiquitin-like modifier activating enzyme 5
Mthfr	17769	ENSMUSG00000029009	5,10-methylenetetrahydrofolate reductase
Acad11	102632	ENSMUSG00000090150	acyl-Coenzyme A dehydrogenase family, member 11
Ppp1r3b	244416	ENSMUSG00000046794	protein phosphatase 1, regulatory (inhibitor) subunit 3B
Cyp2e1	13106	ENSMUSG00000025479	cytochrome P450, family 2, subfamily e, polypeptide 1
Gnmt	14711	ENSMUSG00000002769	glycine N-methyltransferase
Cyp4a14	13119	ENSMUSG00000028715	cytochrome P450, family 4, subfamily a, polypeptide 14
Dio1	13370	ENSMUSG00000034785	deiodinase, iodothyronine, type I
Dbi	13167	ENSMUSG00000026385	diazepam binding inhibitor
Fmo5	14263	ENSMUSG00000028088	flavin containing monooxygenase 5
Mgst1	56615	ENSMUSG00000008540	microsomal glutathione S-transferase 1
Hpd	15445	ENSMUSG00000029445	4-hydroxyphenylpyruvic acid dioxygenase
G6pc	14377	ENSMUSG00000078650	glucose-6-phosphatase, catalytic
Cyp4f14	64385	ENSMUSG00000024292	cytochrome P450, family 4, subfamily f, polypeptide 14
Ppp1r3c	53412	ENSMUSG00000067279	protein phosphatase 1, regulatory (inhibitor) subunit 3C
Ppp1r3g	76487	ENSMUSG00000050423	protein phosphatase 1, regulatory (inhibitor) subunit 3G
**biological process----organic acid metabolic process----GO:0006082**			
C=488;O=15;E=3.63;R=4.13;rawP=2.17e-06;adjP=0.0005			
Cyp2d22	56448	ENSMUSG00000061740	cytochrome P450, family 2, subfamily d, polypeptide 22
Mthfr	17769	ENSMUSG00000029009	5,10-methylenetetrahydrofolate reductase
Glo1	109801	ENSMUSG00000024026	glyoxalase 1
Acad11	102632	ENSMUSG00000090150	acyl-Coenzyme A dehydrogenase family, member 11
Acacb	100705	ENSMUSG00000042010	acetyl-Coenzyme A carboxylase beta
Insig2	72999	ENSMUSG00000003721	insulin induced gene 2
Pnkd	56695	ENSMUSG00000026179	paroxysmal nonkinesiogenic dyskinesia
Agxt	11611	ENSMUSG00000026272	alanine-glyoxylate aminotransferase
Gnmt	14711	ENSMUSG00000002769	glycine N-methyltransferase
Cyp4a14	13119	ENSMUSG00000028715	cytochrome P450, family 4, subfamily a, polypeptide 14
Dbi	13167	ENSMUSG00000026385	diazepam binding inhibitor
Mgst1	56615	ENSMUSG00000008540	microsomal glutathione S-transferase 1
Hpd	15445	ENSMUSG00000029445	4-hydroxyphenylpyruvic acid dioxygenase
G6pc	14377	ENSMUSG00000078650	glucose-6-phosphatase, catalytic
Cyp4f14	64385	ENSMUSG00000024292	cytochrome P450, family 4, subfamily f, polypeptide 14
**biological process----carboxylic acid metabolic process----GO:0019752**			
C=462;O=14;E=3.44;R=4.07;rawP=5.93e-06;adjP=0.0006			
Cyp2d22	56448	ENSMUSG00000061740	cytochrome P450, family 2, subfamily d, polypeptide 22
Mthfr	17769	ENSMUSG00000029009	5,10-methylenetetrahydrofolate reductase
Glo1	109801	ENSMUSG00000024026	glyoxalase 1
Acad11	102632	ENSMUSG00000090150	acyl-Coenzyme A dehydrogenase family, member 11
Acacb	100705	ENSMUSG00000042010	acetyl-Coenzyme A carboxylase beta
Insig2	72999	ENSMUSG00000003721	insulin induced gene 2
Pnkd	56695	ENSMUSG00000026179	paroxysmal nonkinesiogenic dyskinesia
Agxt	11611	ENSMUSG00000026272	alanine-glyoxylate aminotransferase
Gnmt	14711	ENSMUSG00000002769	glycine N-methyltransferase
Cyp4a14	13119	ENSMUSG00000028715	cytochrome P450, family 4, subfamily a, polypeptide 14
Dbi	13167	ENSMUSG00000026385	diazepam binding inhibitor
Mgst1	56615	ENSMUSG00000008540	microsomal glutathione S-transferase 1
Hpd	15445	ENSMUSG00000029445	4-hydroxyphenylpyruvic acid dioxygenase
Cyp4f14	64385	ENSMUSG00000024292	cytochrome P450, family 4, subfamily f, polypeptide 14
**biological process----fatty acid metabolic process----GO:0006631**			
C=177;O=9;E=1.32;R=6.83;rawP=5.83e-06;adjP=0.0006			
Cyp2d22	56448	ENSMUSG00000061740	cytochrome P450, family 2, subfamily d, polypeptide 22
Mthfr	17769	ENSMUSG00000029009	5,10-methylenetetrahydrofolate reductase
Acad11	102632	ENSMUSG00000090150	acyl-Coenzyme A dehydrogenase family, member 11
Acacb	100705	ENSMUSG00000042010	acetyl-Coenzyme A carboxylase beta
Insig2	72999	ENSMUSG00000003721	insulin induced gene 2
Gnmt	14711	ENSMUSG00000002769	glycine N-methyltransferase
Cyp4a14	13119	ENSMUSG00000028715	cytochrome P450, family 4, subfamily a, polypeptide 14
Dbi	13167	ENSMUSG00000026385	diazepam binding inhibitor
Cyp4f14	64385	ENSMUSG00000024292	cytochrome P450, family 4, subfamily f, polypeptide 14
**biological process----monocarboxylic acid metabolic process----GO:0032787**			
C=235;O=10;E=1.75;R=5.72;rawP=8.30e-06;adjP=0.0007			
Cyp2d22	56448	ENSMUSG00000061740	cytochrome P450, family 2, subfamily d, polypeptide 22
Mthfr	17769	ENSMUSG00000029009	5,10-methylenetetrahydrofolate reductase
Acad11	102632	ENSMUSG00000090150	acyl-Coenzyme A dehydrogenase family, member 11
Acacb	100705	ENSMUSG00000042010	acetyl-Coenzyme A carboxylase beta
Insig2	72999	ENSMUSG00000003721	insulin induced gene 2
Agxt	11611	ENSMUSG00000026272	alanine-glyoxylate aminotransferase
Gnmt	14711	ENSMUSG00000002769	glycine N-methyltransferase
Cyp4a14	13119	ENSMUSG00000028715	cytochrome P450, family 4, subfamily a, polypeptide 14
Dbi	13167	ENSMUSG00000026385	diazepam binding inhibitor
Cyp4f14	64385	ENSMUSG00000024292	cytochrome P450, family 4, subfamily f, polypeptide 14
**biological process----oxoacid metabolic process----GO:0043436**			
C=481;O=14;E=3.58;R=3.91;rawP=9.43e-06;adjP=0.0007			
Cyp2d22	56448	ENSMUSG00000061740	cytochrome P450, family 2, subfamily d, polypeptide 22
Mthfr	17769	ENSMUSG00000029009	5,10-methylenetetrahydrofolate reductase
Glo1	109801	ENSMUSG00000024026	glyoxalase 1
Acad11	102632	ENSMUSG00000090150	acyl-Coenzyme A dehydrogenase family, member 11
Acacb	100705	ENSMUSG00000042010	acetyl-Coenzyme A carboxylase beta
Insig2	72999	ENSMUSG00000003721	insulin induced gene 2
Pnkd	56695	ENSMUSG00000026179	paroxysmal nonkinesiogenic dyskinesia
Agxt	11611	ENSMUSG00000026272	alanine-glyoxylate aminotransferase
Gnmt	14711	ENSMUSG00000002769	glycine N-methyltransferase
Cyp4a14	13119	ENSMUSG00000028715	cytochrome P450, family 4, subfamily a, polypeptide 14
Dbi	13167	ENSMUSG00000026385	diazepam binding inhibitor
Mgst1	56615	ENSMUSG00000008540	microsomal glutathione S-transferase 1
Hpd	15445	ENSMUSG00000029445	4-hydroxyphenylpyruvic acid dioxygenase
Cyp4f14	64385	ENSMUSG00000024292	cytochrome P450, family 4, subfamily f, polypeptide 14
**biological process----glycogen metabolic process----GO:0005977**			
C=48;O=5;E=0.36;R=13.99;rawP=2.64e-05;adjP=0.0010			
Ppp1r3b	244416	ENSMUSG00000046794	protein phosphatase 1, regulatory (inhibitor) subunit 3B
G6pc	14377	ENSMUSG00000078650	glucose-6-phosphatase, catalytic
Ppp1r3c	53412	ENSMUSG00000067279	protein phosphatase 1, regulatory (inhibitor) subunit 3C
Ppp1r3g	76487	ENSMUSG00000050423	protein phosphatase 1, regulatory (inhibitor) subunit 3G

16 loci differently marked between A - B and A' - B' against 11,070 marked genes

Significance Level: 0.05, Statistics Test: Hypergeometric, MTC: BH, Minimum: 3

**C**	number of reference genes in the category
**O**	number of genes in the gene set and also in the category
**E**	expected number in the category
**R**	Ratio of enrichment
**rawP**	p value from hypergeometric test (rawP)
**adjP**	p value adjusted by the multiple test adjustment (adjP)

KEGG: no enrichment

Enriched GO categories

**cellular component----endoplasmic reticulum part----GO:0044432**			
C=205;O=3;E=0.28;R=10.57;rawP=0.0025;adjP=0.0258			
Insig2	72999	ENSMUSG0	insulin induced gene 2
Cyp2e1	13106	ENSMUSG0	cytochrome P450, family 2, subfamily e, polypeptide 1
Herpud1	64209	ENSMUSG0	homocysteine-inducible, endoplasmic reticulum stress-inducible, ubiquitin-like domain member 1
**cellular component----endoplasmic reticulum membrane----GO:0005789**			
C=173;O=3;E=0.24;R=12.53;rawP=0.0015;adjP=0.0258			
Insig2	72999	ENSMUSG0	insulin induced gene 2
Cyp2e1	13106	ENSMUSG0	cytochrome P450, family 2, subfamily e, polypeptide 1
Herpud1	64209	ENSMUSG0	homocysteine-inducible, endoplasmic reticulum stress-inducible, ubiquitin-like domain member 1
**cellular component----nuclear outer membrane-endoplasmic reticulum membrane network----GO:0042175**			
C=184;O=3;E=0.25;R=11.78;rawP=0.0018;adjP=0.0258			
Insig2	72999	ENSMUSG0	insulin induced gene 2
Cyp2e1	13106	ENSMUSG0	cytochrome P450, family 2, subfamily e, polypeptide 1
Herpud1	64209	ENSMUSG0	homocysteine-inducible, endoplasmic reticulum stress-inducible, ubiquitin-like domain member 1

Gnmt	14711	ENSMUSG00000002769	glycine N-methyltransferase
**biological process----T cell differentiation----GO:0030217**			
C=76;O=6;E=0.57;R=10.61;rawP=1.98e-05;adjP=0.0010			
Zfp36l1	12192	ENSMUSG00000021127	zinc finger protein 36, C3H type-like 1
Gadd45g	23882	ENSMUSG00000021453	growth arrest and DNA-damage-inducible 45 gamma
Bcl3	12051	ENSMUSG00000053175	B cell leukemia/lymphoma 3
Tgfbr2	21813	ENSMUSG00000032440	transforming growth factor, beta receptor II
Bcl6	12053	ENSMUSG00000022508	B cell leukemia/lymphoma 6
Fzd8	14370	ENSMUSG00000036904	frizzled homolog 8 (Drosophila)
**biological process----cellular glucan metabolic process----GO:0006073**			
C=48;O=5;E=0.36;R=13.99;rawP=2.64e-05;adjP=0.0010			
Ppp1r3b	244416	ENSMUSG00000046794	protein phosphatase 1, regulatory (inhibitor) subunit 3B
G6pc	14377	ENSMUSG00000078650	glucose-6-phosphatase, catalytic
Ppp1r3c	53412	ENSMUSG00000067279	protein phosphatase 1, regulatory (inhibitor) subunit 3C
Ppp1r3g	76487	ENSMUSG00000050423	protein phosphatase 1, regulatory (inhibitor) subunit 3G
Gnmt	14711	ENSMUSG00000002769	glycine N-methyltransferase
**biological process----energy reserve metabolic process----GO:0006112**			
C=49;O=5;E=0.36;R=13.71;rawP=2.92e-05;adjP=0.0010			
Ppp1r3b	244416	ENSMUSG00000046794	protein phosphatase 1, regulatory (inhibitor) subunit 3B
G6pc	14377	ENSMUSG00000078650	glucose-6-phosphatase, catalytic
Ppp1r3c	53412	ENSMUSG00000067279	protein phosphatase 1, regulatory (inhibitor) subunit 3C
Ppp1r3g	76487	ENSMUSG00000050423	protein phosphatase 1, regulatory (inhibitor) subunit 3G
Gnmt	14711	ENSMUSG00000002769	glycine N-methyltransferase
**biological process----glucan metabolic process----GO:0044042**			
C=48;O=5;E=0.36;R=13.99;rawP=2.64e-05;adjP=0.0010			
Ppp1r3b	244416	ENSMUSG00000046794	protein phosphatase 1, regulatory (inhibitor) subunit 3B
G6pc	14377	ENSMUSG00000078650	glucose-6-phosphatase, catalytic
Ppp1r3c	53412	ENSMUSG00000067279	protein phosphatase 1, regulatory (inhibitor) subunit 3C
Ppp1r3g	76487	ENSMUSG00000050423	protein phosphatase 1, regulatory (inhibitor) subunit 3G
Gnmt	14711	ENSMUSG00000002769	glycine N-methyltransferase
**biological process----arachidonic acid metabolic process----GO:0019369**			
C=8;O=3;E=0.06;R=50.38;rawP=2.15e-05;adjP=0.0010			
Cyp4a14	13119	ENSMUSG00000028715	cytochrome P450, family 4, subfamily a, polypeptide 14
Cyp2d22	56448	ENSMUSG00000061740	cytochrome P450, family 2, subfamily d, polypeptide 22
Cyp4f14	64385	ENSMUSG00000024292	cytochrome P450, family 4, subfamily f, polypeptide 14
**biological process----leukocyte activation----GO:0045321**			
C=222;O=9;E=1.65;R=5.45;rawP=3.59e-05;adjP=0.0012			
Bcl6	12053	ENSMUSG00000022508	B cell leukemia/lymphoma 6
Cdkn1a	12575	ENSMUSG00000023067	cyclin-dependent kinase inhibitor 1A (P21)
Bcl3	12051	ENSMUSG00000053175	B cell leukemia/lymphoma 3
Gadd45g	23882	ENSMUSG00000021453	growth arrest and DNA-damage-inducible 45 gamma
Zfp36l1	12192	ENSMUSG00000021127	zinc finger protein 36, C3H type-like 1
Igfbp2	16008	ENSMUSG00000039323	insulin-like growth factor binding protein 2
Tgfbr2	21813	ENSMUSG00000032440	transforming growth factor, beta receptor II
Lbp	16803	ENSMUSG00000016024	lipopolysaccharide binding protein
Fzd8	14370	ENSMUSG00000036904	frizzled homolog 8 (Drosophila)
**biological process----alpha-beta T cell differentiation----GO:0046632**			
C=30;O=4;E=0.22;R=17.91;rawP=6.66e-05;adjP=0.0014			
Gadd45g	23882	ENSMUSG00000021453	growth arrest and DNA-damage-inducible 45 gamma
Bcl3	12051	ENSMUSG00000053175	B cell leukemia/lymphoma 3
Tgfbr2	21813	ENSMUSG00000032440	transforming growth factor, beta receptor II
Bcl6	12053	ENSMUSG00000022508	B cell leukemia/lymphoma 6
**biological process----polysaccharide metabolic process----GO:0005976**			
C=60;O=5;E=0.45;R=11.19;rawP=7.85e-05;adjP=0.0014			
Ppp1r3b	244416	ENSMUSG00000046794	protein phosphatase 1, regulatory (inhibitor) subunit 3B
G6pc	14377	ENSMUSG00000078650	glucose-6-phosphatase, catalytic
Ppp1r3c	53412	ENSMUSG00000067279	protein phosphatase 1, regulatory (inhibitor) subunit 3C
Ppp1r3g	76487	ENSMUSG00000050423	protein phosphatase 1, regulatory (inhibitor) subunit 3G
Gnmt	14711	ENSMUSG00000002769	glycine N-methyltransferase
**biological process----regulation of carbohydrate biosynthetic process----GO:0043255**			
C=36;O=4;E=0.27;R=14.93;rawP=0.0001;adjP=0.0014			
Ppp1r3b	244416	ENSMUSG00000046794	protein phosphatase 1, regulatory (inhibitor) subunit 3B
Ppp1r3c	53412	ENSMUSG00000067279	protein phosphatase 1, regulatory (inhibitor) subunit 3C
Ppp1r3g	76487	ENSMUSG00000050423	protein phosphatase 1, regulatory (inhibitor) subunit 3G
Gnmt	14711	ENSMUSG00000002769	glycine N-methyltransferase
**biological process----T cell activation----GO:0042110**			
C=133;O=7;E=0.99;R=7.07;rawP=5.50e-05;adjP=0.0014			
Zfp36l1	12192	ENSMUSG00000021127	zinc finger protein 36, C3H type-like 1
Gadd45g	23882	ENSMUSG00000021453	growth arrest and DNA-damage-inducible 45 gamma
Bcl3	12051	ENSMUSG00000053175	B cell leukemia/lymphoma 3
Igfbp2	16008	ENSMUSG00000039323	insulin-like growth factor binding protein 2
Tgfbr2	21813	ENSMUSG00000032440	transforming growth factor, beta receptor II
Bcl6	12053	ENSMUSG00000022508	B cell leukemia/lymphoma 6
Fzd8	14370	ENSMUSG00000036904	frizzled homolog 8 (Drosophila)
**biological process----lymphocyte activation----GO:0046649**			
C=190;O=8;E=1.41;R=5.66;rawP=7.72e-05;adjP=0.0014			
Bcl6	12053	ENSMUSG00000022508	B cell leukemia/lymphoma 6
Cdkn1a	12575	ENSMUSG00000023067	cyclin-dependent kinase inhibitor 1A (P21)
Bcl3	12051	ENSMUSG00000053175	B cell leukemia/lymphoma 3
Gadd45g	23882	ENSMUSG00000021453	growth arrest and DNA-damage-inducible 45 gamma
Zfp36l1	12192	ENSMUSG00000021127	zinc finger protein 36, C3H type-like 1
Tgfbr2	21813	ENSMUSG00000032440	transforming growth factor, beta receptor II
Igfbp2	16008	ENSMUSG00000039323	insulin-like growth factor binding protein 2
Fzd8	14370	ENSMUSG00000036904	frizzled homolog 8 (Drosophila)
**biological process----regulation of cellular carbohydrate metabolic process----GO:0010675**			
C=66;O=5;E=0.49;R=10.18;rawP=0.0001;adjP=0.0014			
Ddit4	74747	ENSMUSG00000020108	DNA-damage-inducible transcript 4
Ppp1r3b	244416	ENSMUSG00000046794	protein phosphatase 1, regulatory (inhibitor) subunit 3B
Ppp1r3c	53412	ENSMUSG00000067279	protein phosphatase 1, regulatory (inhibitor) subunit 3C
Ppp1r3g	76487	ENSMUSG00000050423	protein phosphatase 1, regulatory (inhibitor) subunit 3G
Gnmt	14711	ENSMUSG00000002769	glycine N-methyltransferase
**biological process----glycogen catabolic process----GO:0005980**			
C=12;O=3;E=0.09;R=33.58;rawP=8.28e-05;adjP=0.0014			
Ppp1r3b	244416	ENSMUSG00000046794	protein phosphatase 1, regulatory (inhibitor) subunit 3B
G6pc	14377	ENSMUSG00000078650	glucose-6-phosphatase, catalytic
Ppp1r3c	53412	ENSMUSG00000067279	protein phosphatase 1, regulatory (inhibitor) subunit 3C
**biological process----cellular lipid metabolic process----GO:0044255**			
C=422;O=12;E=3.14;R=3.82;rawP=5.64e-05;adjP=0.0014			
Cyp2d22	56448	ENSMUSG00000061740	cytochrome P450, family 2, subfamily d, polypeptide 22
Mthfr	17769	ENSMUSG00000029009	5,10-methylenetetrahydrofolate reductase
Acad11	102632	ENSMUSG00000090150	acyl-Coenzyme A dehydrogenase family, member 11
Acacb	100705	ENSMUSG00000042010	acetyl-Coenzyme A carboxylase beta
Etnk2	214253	ENSMUSG00000070644	ethanolamine kinase 2
Insig2	72999	ENSMUSG00000003721	insulin induced gene 2
Cyp2e1	13106	ENSMUSG00000025479	cytochrome P450, family 2, subfamily e, polypeptide 1
Gnmt	14711	ENSMUSG00000002769	glycine N-methyltransferase
Cyp4a14	13119	ENSMUSG00000028715	cytochrome P450, family 4, subfamily a, polypeptide 14
Dbi	13167	ENSMUSG00000026385	diazepam binding inhibitor
G6pc	14377	ENSMUSG00000078650	glucose-6-phosphatase, catalytic
Cyp4f14	64385	ENSMUSG00000024292	cytochrome P450, family 4, subfamily f, polypeptide 14
**biological process----glucan catabolic process----GO:0009251**			
C=12;O=3;E=0.09;R=33.58;rawP=8.28e-05;adjP=0.0014			
Ppp1r3b	244416	ENSMUSG00000046794	protein phosphatase 1, regulatory (inhibitor) subunit 3B
G6pc	14377	ENSMUSG00000078650	glucose-6-phosphatase, catalytic
Ppp1r3c	53412	ENSMUSG00000067279	protein phosphatase 1, regulatory (inhibitor) subunit 3C
**biological process----polysaccharide catabolic process----GO:0000272**			
C=12;O=3;E=0.09;R=33.58;rawP=8.28e-05;adjP=0.0014			
Ppp1r3b	244416	ENSMUSG00000046794	protein phosphatase 1, regulatory (inhibitor) subunit 3B
G6pc	14377	ENSMUSG00000078650	glucose-6-phosphatase, catalytic
Ppp1r3c	53412	ENSMUSG00000067279	protein phosphatase 1, regulatory (inhibitor) subunit 3C
**biological process----cell activation----GO:0001775**			
C=252;O=9;E=1.88;R=4.80;rawP=9.59e-05;adjP=0.0014			
Bcl6	12053	ENSMUSG00000022508	B cell leukemia/lymphoma 6
Cdkn1a	12575	ENSMUSG00000023067	cyclin-dependent kinase inhibitor 1A (P21)
Bcl3	12051	ENSMUSG00000053175	B cell leukemia/lymphoma 3
Gadd45g	23882	ENSMUSG00000021453	growth arrest and DNA-damage-inducible 45 gamma
Zfp36l1	12192	ENSMUSG00000021127	zinc finger protein 36, C3H type-like 1
Igfbp2	16008	ENSMUSG00000039323	insulin-like growth factor binding protein 2
Tgfbr2	21813	ENSMUSG00000032440	transforming growth factor, beta receptor II

Lbp	16803	ENSMUSG00000016024	lipopolysaccharide binding protein
Fzd8	14370	ENSMUSG00000036904	frizzled homolog 8 (Drosophila)
**biological process----regulation of glucose metabolic process----GO:0010906**			
C=56;O=5;E=0.42;R=11.99;rawP=5.62e-05;adjP=0.0014			
Ddit4	74747	ENSMUSG00000020108	DNA-damage-inducible transcript 4
Ppp1r3b	244416	ENSMUSG00000046794	protein phosphatase 1, regulatory (inhibitor) subunit 3B
Ppp1r3c	53412	ENSMUSG00000067279	protein phosphatase 1, regulatory (inhibitor) subunit 3C
Ppp1r3g	76487	ENSMUSG00000050423	protein phosphatase 1, regulatory (inhibitor) subunit 3G
Gnmt	14711	ENSMUSG00000002769	glycine N-methyltransferase
**biological process----cellular polysaccharide metabolic process----GO:0044264**			
C=57;O=5;E=0.42;R=11.78;rawP=6.12e-05;adjP=0.0014			
Ppp1r3b	244416	ENSMUSG00000046794	protein phosphatase 1, regulatory (inhibitor) subunit 3B
G6pc	14377	ENSMUSG00000078650	glucose-6-phosphatase, catalytic
Ppp1r3c	53412	ENSMUSG00000067279	protein phosphatase 1, regulatory (inhibitor) subunit 3C
Ppp1r3g	76487	ENSMUSG00000050423	protein phosphatase 1, regulatory (inhibitor) subunit 3G
Gnmt	14711	ENSMUSG00000002769	glycine N-methyltransferase
**biological process----cellular carbohydrate catabolic process----GO:0044275**			
C=31;O=4;E=0.23;R=17.33;rawP=7.60e-05;adjP=0.0014			
Ddit4	74747	ENSMUSG00000020108	DNA-damage-inducible transcript 4
Ppp1r3b	244416	ENSMUSG00000046794	protein phosphatase 1, regulatory (inhibitor) subunit 3B
G6pc	14377	ENSMUSG00000078650	glucose-6-phosphatase, catalytic
Ppp1r3c	53412	ENSMUSG00000067279	protein phosphatase 1, regulatory (inhibitor) subunit 3C
**biological process----lymphocyte differentiation----GO:0030098**			
C=104;O=6;E=0.77;R=7.75;rawP=0.0001;adjP=0.0014			
Zfp36l1	12192	ENSMUSG00000021127	zinc finger protein 36, C3H type-like 1
Gadd45g	23882	ENSMUSG00000021453	growth arrest and DNA-damage-inducible 45 gamma
Bcl3	12051	ENSMUSG00000053175	B cell leukemia/lymphoma 3
Tgfbr2	21813	ENSMUSG00000032440	transforming growth factor, beta receptor II
Bcl6	12053	ENSMUSG00000022508	B cell leukemia/lymphoma 6
Fzd8	14370	ENSMUSG00000036904	frizzled homolog 8 (Drosophila)
**biological process----cellular polysaccharide catabolic process----GO:0044247**			
C=12;O=3;E=0.09;R=33.58;rawP=8.28e-05;adjP=0.0014			
Ppp1r3b	244416	ENSMUSG00000046794	protein phosphatase 1, regulatory (inhibitor) subunit 3B
G6pc	14377	ENSMUSG00000078650	glucose-6-phosphatase, catalytic
Ppp1r3c	53412	ENSMUSG00000067279	protein phosphatase 1, regulatory (inhibitor) subunit 3C
**biological process----CD4-positive, alpha-beta T cell differentiation involved in immune response----GO:0002294**			
C=17;O=3;E=0.13;R=23.71;rawP=0.0002;adjP=0.0021			
Gadd45g	23882	ENSMUSG00000021453	growth arrest and DNA-damage-inducible 45 gamma
Bcl3	12051	ENSMUSG00000053175	B cell leukemia/lymphoma 3
Bcl6	12053	ENSMUSG00000022508	B cell leukemia/lymphoma 6
**biological process----positive regulation of leukocyte activation----GO:0002696**			
C=72;O=5;E=0.54;R=9.33;rawP=0.0002;adjP=0.0021			
Cdkn1a	12575	ENSMUSG00000023067	cyclin-dependent kinase inhibitor 1A (P21)
Igfbp2	16008	ENSMUSG00000039323	insulin-like growth factor binding protein 2
Lbp	16803	ENSMUSG00000016024	lipopolysaccharide binding protein
Tgfbr2	21813	ENSMUSG00000032440	transforming growth factor, beta receptor II
Bcl6	12053	ENSMUSG00000022508	B cell leukemia/lymphoma 6
**biological process----lipid metabolic process----GO:0006629**			
C=564;O=13;E=4.20;R=3.10;rawP=0.0002;adjP=0.0021			
Cyp2d22	56448	ENSMUSG00000061740	cytochrome P450, family 2, subfamily d, polypeptide 22
Serpina6	12401	ENSMUSG00000060807	serine (or cysteine) peptidase inhibitor, clade A, member 6
Mthfr	17769	ENSMUSG00000029009	5,10-methylenetetrahydrofolate reductase
Acad11	102632	ENSMUSG00000090150	acyl-Coenzyme A dehydrogenase family, member 11
Acacb	100705	ENSMUSG00000042010	acetyl-Coenzyme A carboxylase beta
Etnk2	214253	ENSMUSG00000070644	ethanolamine kinase 2
Insig2	72999	ENSMUSG00000003721	insulin induced gene 2
Cyp2e1	13106	ENSMUSG00000025479	cytochrome P450, family 2, subfamily e, polypeptide 1
Gnmt	14711	ENSMUSG00000002769	glycine N-methyltransferase
Cyp4a14	13119	ENSMUSG00000028715	cytochrome P450, family 4, subfamily a, polypeptide 14
Dbi	13167	ENSMUSG00000026385	diazepam binding inhibitor
G6pc	14377	ENSMUSG00000078650	glucose-6-phosphatase, catalytic
Cyp4f14	64385	ENSMUSG00000024292	cytochrome P450, family 4, subfamily f, polypeptide 14
**biological process----T-helper cell differentiation----GO:0042093**			
C=17;O=3;E=0.13;R=23.71;rawP=0.0002;adjP=0.0021			
Gadd45g	23882	ENSMUSG00000021453	growth arrest and DNA-damage-inducible 45 gamma
Bcl3	12051	ENSMUSG00000053175	B cell leukemia/lymphoma 3
Bcl6	12053	ENSMUSG00000022508	B cell leukemia/lymphoma 6
**biological process----alpha-beta T cell activation----GO:0046631**			
C=37;O=4;E=0.28;R=14.52;rawP=0.0002;adjP=0.0021			
Gadd45g	23882	ENSMUSG00000021453	growth arrest and DNA-damage-inducible 45 gamma
Bcl3	12051	ENSMUSG00000053175	B cell leukemia/lymphoma 3
Tgfbr2	21813	ENSMUSG00000032440	transforming growth factor, beta receptor II
Bcl6	12053	ENSMUSG00000022508	B cell leukemia/lymphoma 6
**biological process----alpha-beta T cell differentiation involved in immune response----GO:0002293**			
C=17;O=3;E=0.13;R=23.71;rawP=0.0002;adjP=0.0021			
Gadd45g	23882	ENSMUSG00000021453	growth arrest and DNA-damage-inducible 45 gamma
Bcl3	12051	ENSMUSG00000053175	B cell leukemia/lymphoma 3
Bcl6	12053	ENSMUSG00000022508	B cell leukemia/lymphoma 6
**biological process----positive regulation of cell activation----GO:0050867**			
C=75;O=5;E=0.56;R=8.96;rawP=0.0002;adjP=0.0021			
Cdkn1a	12575	ENSMUSG00000023067	cyclin-dependent kinase inhibitor 1A (P21)
Igfbp2	16008	ENSMUSG00000039323	insulin-like growth factor binding protein 2
Lbp	16803	ENSMUSG00000016024	lipopolysaccharide binding protein
Tgfbr2	21813	ENSMUSG00000032440	transforming growth factor, beta receptor II
Bcl6	12053	ENSMUSG00000022508	B cell leukemia/lymphoma 6
**biological process----regulation of generation of precursor metabolites and energy----GO:0043467**			
C=39;O=4;E=0.29;R=13.78;rawP=0.0002;adjP=0.0021			
Ddit4	74747	ENSMUSG00000020108	DNA-damage-inducible transcript 4
Ppp1r3b	244416	ENSMUSG00000046794	protein phosphatase 1, regulatory (inhibitor) subunit 3B
Ppp1r3c	53412	ENSMUSG00000067279	protein phosphatase 1, regulatory (inhibitor) subunit 3C
Ppp1r3g	76487	ENSMUSG00000050423	protein phosphatase 1, regulatory (inhibitor) subunit 3G
**biological process----T cell differentiation involved in immune response----GO:0002292**			
C=17;O=3;E=0.13;R=23.71;rawP=0.0002;adjP=0.0021			
Gadd45g	23882	ENSMUSG00000021453	growth arrest and DNA-damage-inducible 45 gamma
Bcl3	12051	ENSMUSG00000053175	B cell leukemia/lymphoma 3
Bcl6	12053	ENSMUSG00000022508	B cell leukemia/lymphoma 6
**biological process----regulation of carbohydrate metabolic process----GO:0006109**			
C=69;O=5;E=0.51;R=9.73;rawP=0.0002;adjP=0.0021			
Ddit4	74747	ENSMUSG00000020108	DNA-damage-inducible transcript 4
Ppp1r3b	244416	ENSMUSG00000046794	protein phosphatase 1, regulatory (inhibitor) subunit 3B
Ppp1r3c	53412	ENSMUSG00000067279	protein phosphatase 1, regulatory (inhibitor) subunit 3C
Ppp1r3g	76487	ENSMUSG00000050423	protein phosphatase 1, regulatory (inhibitor) subunit 3G
Gnmt	14711	ENSMUSG00000002769	glycine N-methyltransferase
**biological process----glucose metabolic process----GO:0006006**			
C=123;O=6;E=0.92;R=6.55;rawP=0.0003;adjP=0.0031			
Ddit4	74747	ENSMUSG00000020108	DNA-damage-inducible transcript 4
Ppp1r3b	244416	ENSMUSG00000046794	protein phosphatase 1, regulatory (inhibitor) subunit 3B
G6pc	14377	ENSMUSG00000078650	glucose-6-phosphatase, catalytic
Ppp1r3c	53412	ENSMUSG00000067279	protein phosphatase 1, regulatory (inhibitor) subunit 3C
Ppp1r3g	76487	ENSMUSG00000050423	protein phosphatase 1, regulatory (inhibitor) subunit 3G
Gnmt	14711	ENSMUSG00000002769	glycine N-methyltransferase
**molecular function----arachidonic acid monooxygenase activity----GO:0008391**			
C=5;O=3;E=0.04;R=83.55;rawP=3.50e-06;adjP=0.0003			
Cyp4a14	13119	ENSMUSG00000028715	cytochrome P450, family 4, subfamily a, polypeptide 14
Cyp2d22	56448	ENSMUSG00000061740	cytochrome P450, family 2, subfamily d, polypeptide 22
Cyp4f14	64385	ENSMUSG00000024292	cytochrome P450, family 4, subfamily f, polypeptide 14
**molecular function----monooxygenase activity----GO:0004497**			
C=50;O=5;E=0.36;R=13.92;rawP=2.70e-05;adjP=0.0011			
Cyp4a14	13119	ENSMUSG00000028715	cytochrome P450, family 4, subfamily a, polypeptide 14
Cyp2d22	56448	ENSMUSG00000061740	cytochrome P450, family 2, subfamily d, polypeptide 22
Fmo5	14263	ENSMUSG00000028088	flavin containing monooxygenase 5
Cyp4f14	64385	ENSMUSG00000024292	cytochrome P450, family 4, subfamily f, polypeptide 14
Cyp2e1	13106	ENSMUSG00000025479	cytochrome P450, family 2, subfamily e, polypeptide 1
**molecular function----carboxylic acid binding----GO:0031406**			
C=103;O=6;E=0.74;R=8.11;rawP=9.03e-05;adjP=0.0024			
Mthfr	17769	ENSMUSG00000029009	5,10-methylenetetrahydrofolate reductase
Mgst1	56615	ENSMUSG00000008540	microsomal glutathione S-transferase 1
Acacb	100705	ENSMUSG00000042010	acetyl-Coenzyme A carboxylase beta
Cyp4f14	64385	ENSMUSG00000024292	cytochrome P450, family 4, subfamily f, polypeptide 14

Agxt	11611	ENSMUSG00000026272	alanine-glyoxylate aminotransferase
Gnmt	14711	ENSMUSG00000002769	glycine N-methyltransferase
**molecular function----endoribonuclease activity, producing 5'-phosphomonoesters----GO:0016891**			
C=18;O=3;E=0.13;R=23.21;rawP=0.0003;adjP=0.0060			
Elac1	114615	ENSMUSG00000036941	elaC homolog 1 (E. coli)
Rpp21	67676	ENSMUSG00000024446	ribonuclease P 21 subunit (human)
Pop4	66161	ENSMUSG00000030423	processing of precursor 4, ribonuclease P/MRP family, (S. cerevisiae)
**molecular function----oxidoreductase activity----GO:0016491**			
C=456;O=11;E=3.27;R=3.36;rawP=0.0004;adjP=0.0062			
Cyp2d22	56448	ENSMUSG00000061740	cytochrome P450, family 2, subfamily d, polypeptide 22
Uba5	66663	ENSMUSG00000032557	ubiquitin-like modifier activating enzyme 5
Mthfr	17769	ENSMUSG00000029009	5,10-methylenetetrahydrofolate reductase
Acad11	102632	ENSMUSG00000090150	acyl-Coenzyme A dehydrogenase family, member 11
Cyp2e1	13106	ENSMUSG00000025479	cytochrome P450, family 2, subfamily e, polypeptide 1
Cyp4a14	13119	ENSMUSG00000028715	cytochrome P450, family 4, subfamily a, polypeptide 14
Fmo5	14263	ENSMUSG00000028088	flavin containing monooxygenase 5
Dio1	13370	ENSMUSG00000034785	deiodinase, iodothyronine, type I
Mgst1	56615	ENSMUSG00000008540	microsomal glutathione S-transferase 1
Hpd	15445	ENSMUSG00000029445	4-hydroxyphenylpyruvic acid dioxygenase
Cyp4f14	64385	ENSMUSG00000024292	cytochrome P450, family 4, subfamily f, polypeptide 14
**molecular function----endonuclease activity, active with either ribo- or deoxyribonucleic acids and producing 5'-**			
C=23;O=3;E=0.17;R=18.16;rawP=0.0006;adjP=0.0062			
Elac1	114615	ENSMUSG00000036941	elaC homolog 1 (E. coli)
Rpp21	67676	ENSMUSG00000024446	ribonuclease P 21 subunit (human)
Pop4	66161	ENSMUSG00000030423	processing of precursor 4, ribonuclease P/MRP family, (S. cerevisiae)
**molecular function----oxidoreductase activity, acting on paired donors, with incorporation or reduction of**			
C=98;O=5;E=0.70;R=7.10;rawP=0.0007;adjP=0.0062			
Cyp4a14	13119	ENSMUSG00000028715	cytochrome P450, family 4, subfamily a, polypeptide 14
Cyp2d22	56448	ENSMUSG00000061740	cytochrome P450, family 2, subfamily d, polypeptide 22
Fmo5	14263	ENSMUSG00000028088	flavin containing monooxygenase 5
Cyp4f14	64385	ENSMUSG00000024292	cytochrome P450, family 4, subfamily f, polypeptide 14
Cyp2e1	13106	ENSMUSG00000025479	cytochrome P450, family 2, subfamily e, polypeptide 1
**molecular function----endoribonuclease activity----GO:0004521**			
C=24;O=3;E=0.17;R=17.41;rawP=0.0006;adjP=0.0062			
Elac1	114615	ENSMUSG00000036941	elaC homolog 1 (E. coli)
Rpp21	67676	ENSMUSG00000024446	ribonuclease P 21 subunit (human)
Pop4	66161	ENSMUSG00000030423	processing of precursor 4, ribonuclease P/MRP family, (S. cerevisiae)
**molecular function----amino acid binding----GO:0016597**			
C=57;O=4;E=0.41;R=9.77;rawP=0.0007;adjP=0.0062			
Mthfr	17769	ENSMUSG00000029009	5,10-methylenetetrahydrofolate reductase
Mgst1	56615	ENSMUSG00000008540	microsomal glutathione S-transferase 1
Agxt	11611	ENSMUSG00000026272	alanine-glyoxylate aminotransferase
Gnmt	14711	ENSMUSG00000002769	glycine N-methyltransferase
**molecular function----modified amino acid binding----GO:0072341**			
C=26;O=3;E=0.19;R=16.07;rawP=0.0008;adjP=0.0064			
Mthfr	17769	ENSMUSG00000029009	5,10-methylenetetrahydrofolate reductase
Mgst1	56615	ENSMUSG00000008540	microsomal glutathione S-transferase 1
Gnmt	14711	ENSMUSG00000002769	glycine N-methyltransferase
**molecular function----heme binding----GO:0020037**			
C=60;O=4;E=0.43;R=9.28;rawP=0.0009;adjP=0.0065			
Cyp4a14	13119	ENSMUSG00000028715	cytochrome P450, family 4, subfamily a, polypeptide 14
Cyp2d22	56448	ENSMUSG00000061740	cytochrome P450, family 2, subfamily d, polypeptide 22
Cyp4f14	64385	ENSMUSG00000024292	cytochrome P450, family 4, subfamily f, polypeptide 14
Cyp2e1	13106	ENSMUSG00000025479	cytochrome P450, family 2, subfamily e, polypeptide 1
**molecular function----serine-type endopeptidase inhibitor activity----GO:0004867**			
C=29;O=3;E=0.21;R=14.40;rawP=0.0011;adjP=0.0073			
Serpina3k	20714	ENSMUSG00000058207	serine (or cysteine) peptidase inhibitor, clade A, member 3K
Serpina3m	20717	ENSMUSG00000079012	serine (or cysteine) peptidase inhibitor, clade A, member 3M
Serpina6	12401	ENSMUSG00000060807	serine (or cysteine) peptidase inhibitor, clade A, member 6
**molecular function----tetrapyrrole binding----GO:0046906**			
C=66;O=4;E=0.47;R=8.44;rawP=0.0013;adjP=0.0080			
Cyp4a14	13119	ENSMUSG00000028715	cytochrome P450, family 4, subfamily a, polypeptide 14
Cyp2d22	56448	ENSMUSG00000061740	cytochrome P450, family 2, subfamily d, polypeptide 22
Cyp4f14	64385	ENSMUSG00000024292	cytochrome P450, family 4, subfamily f, polypeptide 14
Cyp2e1	13106	ENSMUSG00000025479	cytochrome P450, family 2, subfamily e, polypeptide 1
**molecular function----ribonuclease activity----GO:0004540**			
C=36;O=3;E=0.26;R=11.60;rawP=0.0021;adjP=0.0120			
Elac1	114615	ENSMUSG00000036941	elaC homolog 1 (E. coli)
Rpp21	67676	ENSMUSG00000024446	ribonuclease P 21 subunit (human)
Pop4	66161	ENSMUSG00000030423	processing of precursor 4, ribonuclease P/MRP family, (S. cerevisiae)
**molecular function----iron ion binding----GO:0005506**			
C=81;O=4;E=0.58;R=6.88;rawP=0.0027;adjP=0.0144			
Cyp4a14	13119	ENSMUSG00000028715	cytochrome P450, family 4, subfamily a, polypeptide 14
Slc11a2	18174	ENSMUSG00000023030	solute carrier family 11 (proton-coupled divalent metal ion transporters), member 2
Cyp4f14	64385	ENSMUSG00000024292	cytochrome P450, family 4, subfamily f, polypeptide 14
Cyp2e1	13106	ENSMUSG00000025479	cytochrome P450, family 2, subfamily e, polypeptide 1
**molecular function----cofactor binding----GO:0048037**			
C=198;O=6;E=1.42;R=4.22;rawP=0.0029;adjP=0.0145			
Uba5	66663	ENSMUSG00000032557	ubiquitin-like modifier activating enzyme 5
Fmo5	14263	ENSMUSG00000028088	flavin containing monooxygenase 5
Mthfr	17769	ENSMUSG00000029009	5,10-methylenetetrahydrofolate reductase
Dbi	13167	ENSMUSG00000026385	diazepam binding inhibitor
Acad11	102632	ENSMUSG00000090150	acyl-Coenzyme A dehydrogenase family, member 11
Agxt	11611	ENSMUSG00000026272	alanine-glyoxylate aminotransferase
**molecular function----peptidase inhibitor activity----GO:0030414**			
C=56;O=3;E=0.40;R=7.46;rawP=0.0075;adjP=0.0333			
Serpina3k	20714	ENSMUSG00000058207	serine (or cysteine) peptidase inhibitor, clade A, member 3K
Serpina3m	20717	ENSMUSG00000079012	serine (or cysteine) peptidase inhibitor, clade A, member 3M
Serpina6	12401	ENSMUSG00000060807	serine (or cysteine) peptidase inhibitor, clade A, member 6
**molecular function----endopeptidase inhibitor activity----GO:0004866**			
C=56;O=3;E=0.40;R=7.46;rawP=0.0075;adjP=0.0333			
Serpina3k	20714	ENSMUSG00000058207	serine (or cysteine) peptidase inhibitor, clade A, member 3K
Serpina3m	20717	ENSMUSG00000079012	serine (or cysteine) peptidase inhibitor, clade A, member 3M
Serpina6	12401	ENSMUSG00000060807	serine (or cysteine) peptidase inhibitor, clade A, member 6
**molecular function----flavin adenine dinucleotide binding----GO:0050660**			
C=57;O=3;E=0.41;R=7.33;rawP=0.0079;adjP=0.0333			
Fmo5	14263	ENSMUSG00000028088	flavin containing monooxygenase 5
Mthfr	17769	ENSMUSG00000029009	5,10-methylenetetrahydrofolate reductase
Acad11	102632	ENSMUSG00000090150	acyl-Coenzyme A dehydrogenase family, member 11
**molecular function----endopeptidase regulator activity----GO:0061135**			
C=61;O=3;E=0.44;R=6.85;rawP=0.0095;adjP=0.0380			
Serpina3k	20714	ENSMUSG00000058207	serine (or cysteine) peptidase inhibitor, clade A, member 3K
Serpina3m	20717	ENSMUSG00000079012	serine (or cysteine) peptidase inhibitor, clade A, member 3M
Serpina6	12401	ENSMUSG00000060807	serine (or cysteine) peptidase inhibitor, clade A, member 6
**molecular function----endonuclease activity----GO:0004519**			
C=63;O=3;E=0.45;R=6.63;rawP=0.0103;adjP=0.0392			
Elac1	114615	ENSMUSG00000036941	elaC homolog 1 (E. coli)
Rpp21	67676	ENSMUSG00000024446	ribonuclease P 21 subunit (human)
Pop4	66161	ENSMUSG00000030423	processing of precursor 4, ribonuclease P/MRP family, (S. cerevisiae)
**molecular function----hydrolase activity, acting on ester bonds----GO:0016788**			
C=441;O=8;E=3.17;R=2.53;rawP=0.0132;adjP=0.0440			
Ppp1r3b	244416	ENSMUSG00000046794	protein phosphatase 1, regulatory (inhibitor) subunit 3B
Elac1	114615	ENSMUSG00000036941	elaC homolog 1 (E. coli)
Rpp21	67676	ENSMUSG00000024446	ribonuclease P 21 subunit (human)
G6pc	14377	ENSMUSG00000078650	glucose-6-phosphatase, catalytic
Dusp1	19252	ENSMUSG00000024190	dual specificity phosphatase 1
Pnkd	56695	ENSMUSG00000026179	paroxysmal nonkinesiogenic dyskinesia
Ppp1r3c	53412	ENSMUSG00000067279	protein phosphatase 1, regulatory (inhibitor) subunit 3C
Pop4	66161	ENSMUSG00000030423	processing of precursor 4, ribonuclease P/MRP family, (S. cerevisiae)
**molecular function----enzyme inhibitor activity----GO:0004857**			
C=127;O=4;E=0.91;R=4.39;rawP=0.0130;adjP=0.0440			
Cdkn1a	12575	ENSMUSG00000023067	cyclin-dependent kinase inhibitor 1A (P21)

Serpina3k	20714	ENSMUSG00000058207	serine (or cysteine) peptidase inhibitor, clade A, member 3K
Serpina3m	20717	ENSMUSG00000079012	serine (or cysteine) peptidase inhibitor, clade A, member 3M
Serpina6	12401	ENSMUSG00000060807	serine (or cysteine) peptidase inhibitor, clade A, member 6
**molecular function----electron carrier activity----GO:0009055**			
C=68;O=3;E=0.49;R=6.14;rawP=0.0127;adjP=0.0440			
Cyp4a14	13119	ENSMUSG00000028715	cytochrome P450, family 4, subfamily a, polypeptide 14
Cyp4f14	64385	ENSMUSG00000024292	cytochrome P450, family 4, subfamily f, polypeptide 14
Cyp2e1	13106	ENSMUSG00000025479	cytochrome P450, family 2, subfamily e, polypeptide 1
